# A replication fork determinant for the establishment of sister chromatid cohesion

**DOI:** 10.1016/j.cell.2022.12.044

**Published:** 2023-01-23

**Authors:** Masashi Minamino, Céline Bouchoux, Berta Canal, John F.X. Diffley, Frank Uhlmann

**Affiliations:** 1 Chromosome Segregation Laboratory, The Francis Crick Institute, London NW1 1AT, UK; 2 Chromosome Replication Laboratory, The Francis Crick Institute, London NW1 1AT, UK

## Abstract

Concomitant with DNA replication, the chromosomal cohesin complex establishes cohesion between newly replicated sister chromatids. Cohesion establishment requires acetylation of conserved cohesin lysine residues by Eco1 acetyltransferase. Here, we explore how cohesin acetylation is linked to DNA replication. Biochemical reconstitution of replication-coupled cohesin acetylation reveals that transient DNA structures, which form during DNA replication, control the acetylation reaction. As polymerases complete lagging strand replication, strand displacement synthesis produces DNA flaps that are trimmed to result in nicked double-stranded DNA. Both flaps and nicks stimulate cohesin acetylation, while subsequent nick ligation to complete Okazaki fragment maturation terminates the acetylation reaction. A flapped or nicked DNA substrate constitutes a transient molecular clue that directs cohesin acetylation to a window behind the replication fork, next to where cohesin likely entraps both sister chromatids. Our results provide an explanation for how DNA replication is linked to sister chromatid cohesion establishment.

## Graphical abstract

**Graphical abstract:**
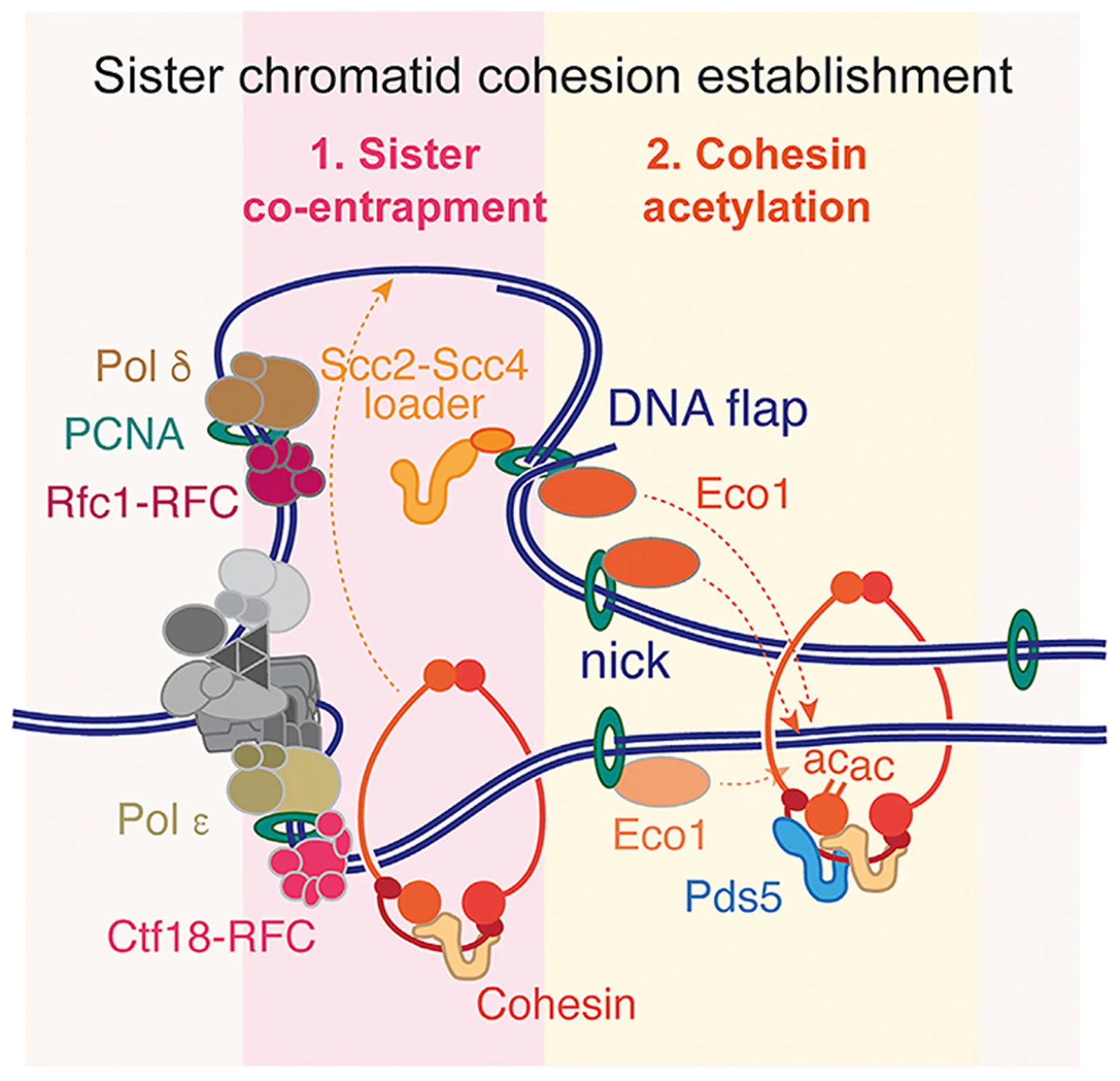

## Introduction

The process of eukaryotic chromosome replication is increasingly well understood.^1–3^ A multitude of protein components come together to form a replisome that achieves processive, high-fidelity duplication of the genome. We are also beginning to understand how epigenetic information, encoded in histone variants and modifications, is maintained and inherited by both sister chromatids.^4^ Faithful chromosome segregation to daughter cells involves an additional critical feature, namely that the two newly synthesized replication products remain connected to one another. This process, known as sister chromatid cohesion, allows the cell division machinery to recognize replication products for faithful segregation into daughter cells during cell divisions.^5–7^ Sister chromatid cohesion is mediated by cohesin, a ring-shaped protein complex that topologically entraps the two sister DNAs.^8,9^

Cohesin is loaded onto DNA already before DNA replication. When the replisome encounters cohesin, two events must take place. First, cohesin transitions from entrapping one DNA to co-entrapping two DNAs, the two sister chromatids. Sister chromatid co-entrapment could arise when replication forks pass through cohesin rings, while those remain topologically closed.^10^ Alternatively, cohesin might dissociate from DNA as the fork approaches, then newly embrace both replication products behind the fork. Sequential cohesin capture of double-stranded DNA (dsDNA) (as present on the leading strand), followed by single-stranded DNA (ssDNA) (characteristic of the lagging strand), could underly new co-entrapment of replication products.^9^ Fork passage through cohesin rings and new co-entrapment behind the fork are not mutually exclusive. The second essential event during sister chromatid cohesin establishment is replication-coupled acetylation of the cohesin subunit Smc3 by the Eco1 acetyltransferase,^11–13^ the subject of our present study.

Before DNA replication, cohesin dynamically loads and unloads from chromosomes.^14–16^ Loading is catalyzed by a “cohesin loader,” consisting of Scc2 and Scc4 subunits, which transiently associates with cohesin. Unloading in turn requires cohesin’s Pds5 subunit, in conjunction with the unloading factor Wapl. Scc2 and Pds5 are structurally similar,^17–19^ and only one or the other alternatingly becomes part of the cohesin complex.^20^ Both Scc2-dependent loading and Pds5-dependent unloading depend on ATP hydrolysis by cohesin’s Smc1 and Smc3 ATPase heads.^20,21^ Loading and unloading further depend on two conserved lysine residues located on the Smc3 ATPase (K112 and K113 in budding yeast), the targets of Eco1. Recent structural insight revealed how the two lysines provide key contacts with DNA and with Scc2 to form an ATP-bound DNA gripping intermediate during cohesin loading onto DNA.^22–24^

Smc3 acetylation stabilizes sister chromatid cohesion as it stops Pds5-Wapl-mediated cohesin unloading, presumably by preventing the formation of a similar ATP-bound DNA unloading intermediate.^11,15,16,25^ However, if Smc3 is acetylated too early, cohesin will be unable to load onto DNA. Acetylated too late, sister chromatid cohesion will be unstable. These considerations explain why cohesin acetylation is tightly linked to DNA replication^11,12^; however, the molecular basis for this link is unknown. Eco1 is transcriptionally upregulated just before the onset of S phase, but Eco1 overexpression throughout all cell cycle stages does not alter the kinetics of cohesin acetylation.^26^ A hint as to how cohesin acetylation is linked to DNA replication comes from an essential proliferating cell nuclear antigen (PCNA) interacting peptide (PIP) box that is found in yeast Eco1.^27,28^ Higher eukaryotes typically contain two Eco1 orthologs, Esco1 and Esco2, that share roles in cohesin acetylation and cohesion establishment. Of these, Esco2 contains an obligate PIP box that promotes replication-coupled cohesin acetylation.^29–33^ However, PCNA is an abundant protein that plays numerous roles, both during and outside of S phase.^34^ Whether and how PCNA helps to define cohesin’s acetylation timing therefore remains unknown.

Here, we investigate replication-coupled cohesin acetylation. We first explore whether posttranslational modifications of Eco1,^35^ or the counteracting cohesin deacetylase Hos1,^26,36^ impose temporal control but find no evidence for those scenarios. We then proceed to biochemically reconstitute replication-coupled cohesin acetylation. These experiments reveal that cohesin acetylation is promoted by DNA structures, flaps or nicks, that transiently form during the maturation of lagging strand DNA synthesis. Fully replicated and ligated DNA no longer supports acetylation. Incorporating previous knowledge about where cohesin likely entraps both sister chromatids, these observations allow us to propose a model for sister chromatid cohesion establishment at DNA replication forks.

## Results

### Cell cycle kinases and the S phase-specificity of cohesin acetylation

**Figure 1:**
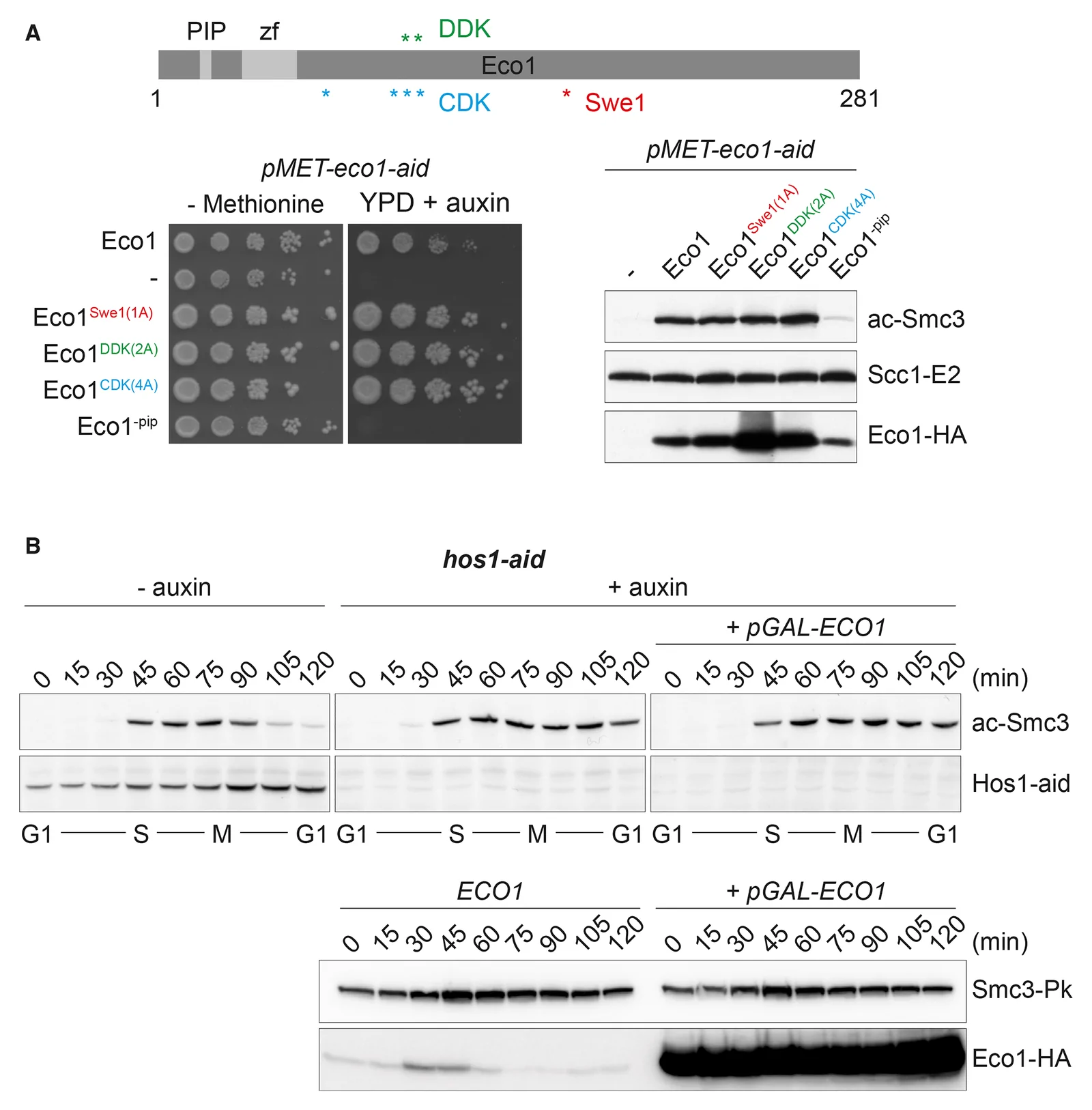
S phase specificity of cohesin acetylation (A) Schematic of Eco1, highlighting its PCNA interacting peptide (PIP) box motif, zinc finger (zf), as well as predicted recognition sites for cell cycle kinases. Viability of cells lacking subsets of these sites was investigated in a strain background in which endogenous Eco1 was depleted by promoter shut-off and auxin-mediated degradation. Eco1 levels were assessed in G2/M-arrested cells by immunoblotting, Smc3 acetylation was monitored using an antibody specific for acetylated Smc3.^11^ Mutated phosphorylation sites were: Swe1 Y164A, DDK S98A-S104A, and CDK S67A-T94A-S99AS-105A. See also Figure S1A for confirmation of Eco1 phosphorylation by Swe1 and Figure S1B confirming unaltered Smc3 acetylation kinetics. (B) Hos1 does not regulate Smc3 acetylation timing. Hos1 was depleted by auxin-mediated degradation and cell cycle-dependent Smc3 acetylation monitored in cells that additionally overexpressed Eco1. Eco1-HA levels following overexpression were compared side-by-side with a strain carrying the same HA epitope tag fused to endogenous Eco1. See also Figure S1C for flow cytometry analyses confirming cell cycle synchrony and Figure S2, demonstrating that Smc3 acetylation is no longer possible after completion of S phase.

Eco1 is a known target of the cyclin-dependent kinase (CDK) and Dbf4-dependent kinase (DDK) cell cycle kinases.^35^ Both CDK and DDK kinase activities increase during S phase to trigger replication origin firing. Although strains carrying CDK phosphorylation site mutations in Eco1 are viable,^37^ it is unknown whether CDK or DDK phosphorylation regulate Eco1 acetyltransferase activity. In addition, mass spectrometry analysis revealed Eco1 phosphorylation on tyrosine 164,^38^ and we confirmed that Eco1 is indeed a target of the budding yeast cell cycle tyrosine kinase Swe1 (Figure S1A). To address whether any of these phosphorylation events regulate Eco1 activity, we assessed Smc3 acetylation in strains in which the phosphorylation sites for each of these kinases were mutated to alanines. The respective non-phosphorylatable Eco1 variants were all proficient in supporting cell growth (Figure 1A). Consistent with the idea that CDK and DDK phosphorylation control an Eco1 phosphodegron,^35^ Eco1 levels were higher in the absence of CDK or DDK phosphorylation sites. However, Smc3 acetylation levels following S phase in all phosphosite mutant strains were indistinguishable from wild type (Figure 1A). Furthermore, the timing of cohesin acetylation remained closely coupled to DNA replication in all cases (Figure S1B). In contrast to the phosphosite mutants, an Eco1 variant in which two PIP box residues were mutated to alanine did not support cell growth nor Smc3 acetylation. We conclude that replication-coupled cohesin acetylation is independent of Eco1 phosphorylation by cell cycle kinases.

### The Hos1 cohesin deacetylase

Cohesin acetylation is a reversible reaction. The Hos1 deacetylase (HDAC8 in humans) erases Smc3 acetylation when cohesin dissociates from DNA in anaphase, which recycles cohesin for loading onto chromosomes during the subsequent G1 phase.^26,36,39^ Might Hos1 restrict cohesin acetylation by counteracting Eco1 until the onset of S phase? To test this possibility, we created a yeast strain from which we could conditionally deplete Hos1 using an auxin-inducible degron.^40^ Hos1 was efficiently depleted by using this strategy, as cohesin deacetylation was no longer observed in anaphase. However, lack of Hos1 did not result in premature Smc3 acetylation (Figures 1B and S1C). Even if Eco1 was overexpressed to markedly increased levels in the absence of Hos1, acetylation remained temporally coupled to DNA replication. This suggests that cohesin acetylation is linked to DNA replication by a yet unknown mechanism that does not involve the Hos1 cohesin deacetylase.

We also investigated whether cohesin acetylation is restricted to ongoing DNA replication or whether Eco1’s acetylation proficiency commences during DNA replication and then persists at later cell cycle stages. To address this, we utilized cells lacking Eco1 and kept alive by the absence of Wapl (*eco1Δ wpl1*Δ).^11^ We synchronized *eco1Δ wpl1*Δ cells to pass through S phase into a G2/M arrest without cohesin acetylation. Eco1 was then added back by expression from a galactose-inducible promoter. Although Eco1 levels soon exceed those observed in wild-type cells, no Smc3 acetylation was detected (Figure S2). These results imply that the cohesin acetylation reaction only takes place in the context of DNA replication.

### Biochemical reconstitution of replication-coupled cohesin acetylation

**Figure 2:**
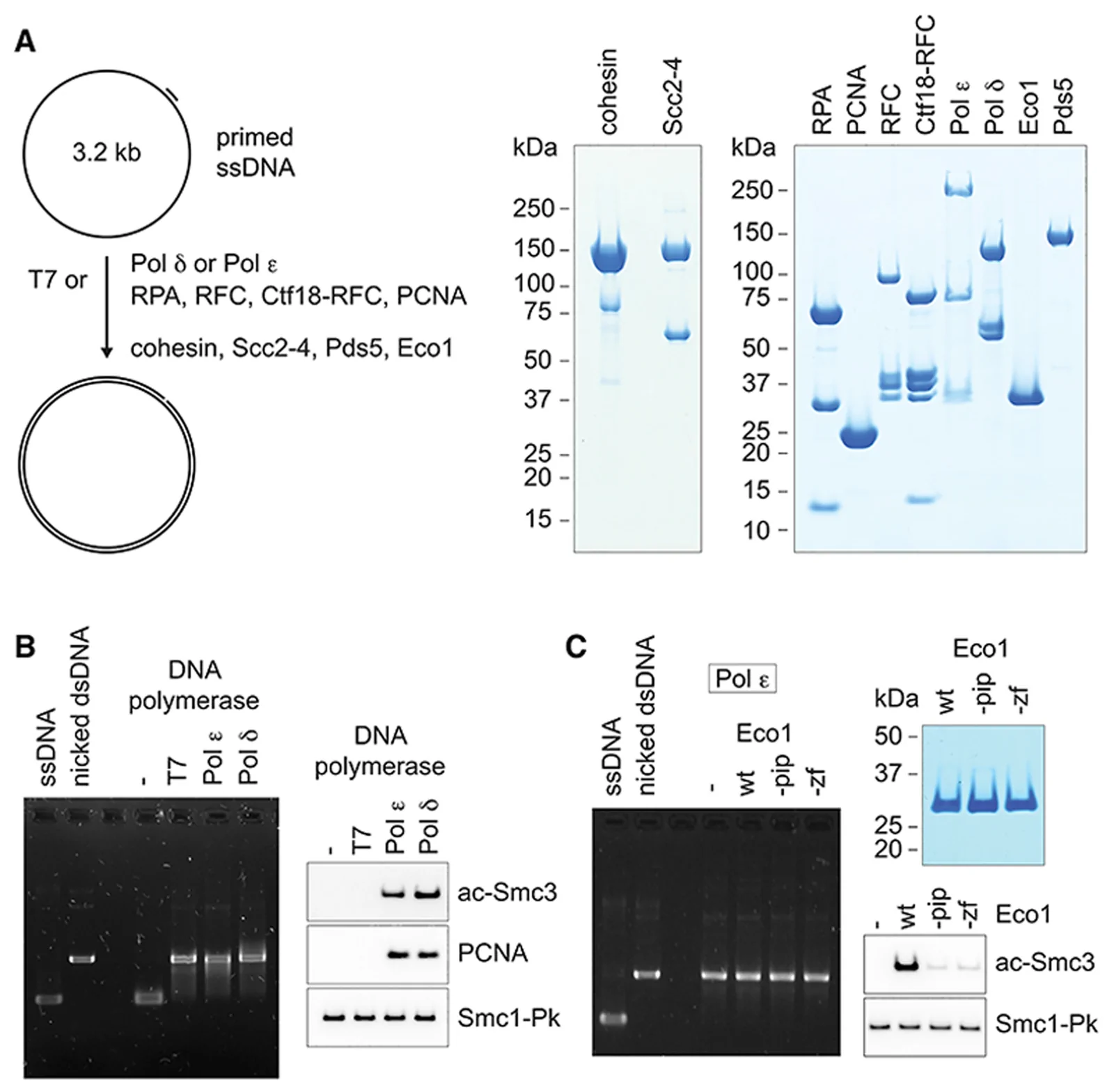
Biochemical reconstitution of replication-coupled cohesin acetylation (A) Schematic of the primed ssDNA replication assay in the presence of cohesin and cohesion establishment factors. Purified cohesin, the Scc2-Scc4 cohesin loader (Scc2-4), RPA, PCNA, RFC, Ctf18-RFC, DNA polymerase ε (Pol ε), DNA polymerase δ (Pol δ), Eco1, and Pds5 were analyzed by SDS-PAGE followed by Coomassie blue staining. (B) Replication-coupled cohesin acetylation. Gel image of the DNA replication products formed by 0.75 U/μL T7 DNA polymerase, 15 nM Pol ε, or 15 nM of Pol δ during the 12-min cohesin acetylation reaction. The single-stranded template DNA and enzymatically nicked double-stranded plasmid DNA were loaded alongside as a reference. Smc3 acetylation and PCNA were detected by immunoblotting, a Pk epitope-tag on the cohesin subunit Smc1 served as a loading control. (C) PIP box- and zinc finger-dependent cohesin acetylation. Gel image of the DNA replication products synthesized by Pol ε and immunoblot to analyze Smc3 acetylation by 70 nM of either wild-type Eco1, PIP box mutant (Eco1^−pip^), or zinc-finger mutant Eco1 (Eco1^−zf^). Incubation was for 60 min. SDS-PAGE analysis, followed by Coomassie blue staining, of purified wild-type and mutant Eco1 is shown.

To understand how Eco1 activity is linked to DNA replication, we set out to biochemically reconstitute replication-coupled cohesin acetylation using budding yeast proteins. PCNA is best known for its role in replication elongation reactions. We therefore used purified PCNA, together with Rfc1-RFC (replication factor C) and Ctf18-RFC as PCNA loaders, ssDNA binding protein RPA, as well as either DNA polymerases δ or ε to elongate a primer annealed to a circular ssDNA template (Figure 2A).^41^ As a control, we used T7 phage DNA polymerase to replicate the same template in the absence of PCNA. Purified budding yeast cohesin, the Scc2-Scc4 cohesin loader, Pds5, and Eco1 were included in all reactions.^28,42^ Replication products were visualized by agarose gel electrophoresis, confirming conversion of the single-stranded template to double-stranded products by all three DNA polymerases. Smc3 acetylation was readily detected in replication reactions using polymerases δ or ε, together with PCNA and accessory factors, but not in reactions with T7 DNA polymerase (Figure 2B). To investigate whether replication-coupled cohesin acetylation seen with eukaryotic replication proteins adheres to known *in vivo* requirements, we repeated the above reaction using Eco1 with a mutated PIP box (Eco1^−pip^)^27,28^ or with two zinc-coordinating residues replaced by alanines (Eco1^−zf^).^43^ Smc3 acetylation was disrupted by the PIP box and zinc-finger mutations (Figure 2C). These results suggest that replication-coupled cohesin acetylation can be reconstituted with purified yeast replication factors *in vitro*, recapitulating known *in vivo* characteristics.

### Cohesin acetylation follows completion of DNA synthesis

**Figure 3:**
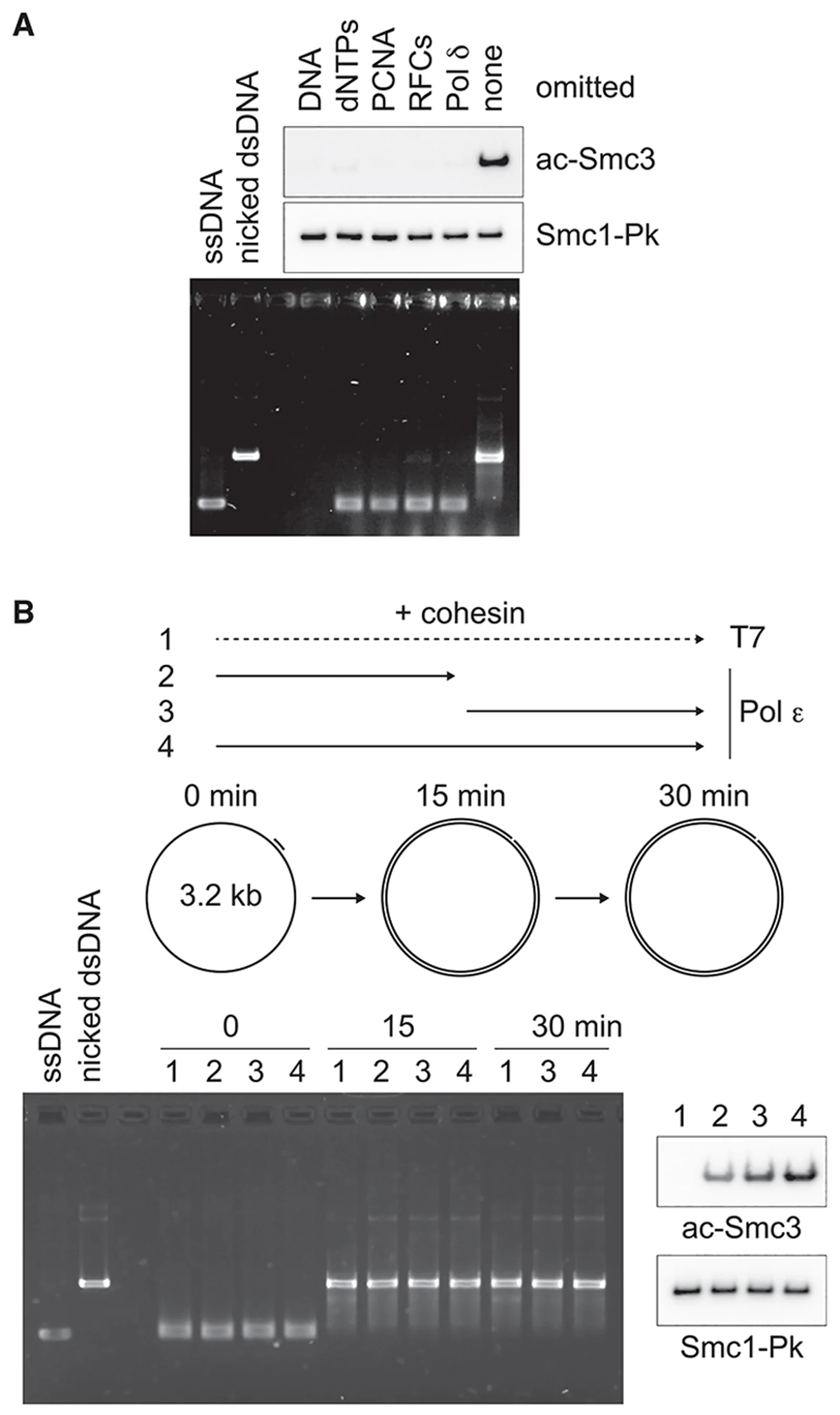
Cohesin acetylation following completion of DNA synthesis (A) DNA synthesis is required for cohesin acetylation. Gel image of the DNA replication products in a primer extension reaction using Pol δ, and immunoblot analysis of Smc3 acetylation in reactions omitting the indicated components. (B) Replication products, rather than ongoing replication, stimulate cohesin acetylation. Schematic of the experiment, indicating the time intervals when cohesin and cohesion establishment factors were added to a Pol ε-mediated primer extension reaction. T7 DNA polymerase served as a control. Gel image of the DNA replication products at the indicated time points during incubation, as well as immunoblot analysis of Smc3 acetylation are shown. The Pk epitope-tag on the cohesin subunit Smc1 served as a loading control. See also Figure S3 for time course analyses suggesting that cohesin acetylation continues after completion of DNA replication.

To gain insight into the mechanism of replication-coupled cohesin acetylation, we repeated the primer elongation reaction, leaving out one component at a time. Acetylation was lost in the absence of PCNA, consistent with its expected role (Figure 3A), but also when any other replication protein was omitted. Even if all proteins were included, but dNTPs were lacking, Smc3 acetylation remained undetectable. The latter result suggests that combining all the eukaryotic replication proteins is insufficient and that DNA synthesis is required as part of the cohesin acetylation reaction.

We next followed cohesin acetylation kinetics, relative to the progression of DNA synthesis. PCNA loading onto DNA occurred within the first minutes, after which DNA replication by Pol ε was complete within 12 min of incubation. Cohesin acetylation became detectable during this 12-min interval but continued to rise until at least 60 min (Figure S3). Similarly, cohesin acetylation continued to rise following complete ssDNA to dsDNA conversion by Pol δ, whereas slow strand displacement DNA synthesis continued in this case. The comparison of replication and acetylation timings opened the possibility that Eco1 acetylates cohesin following completion of DNA synthesis.

To clarify when Eco1 acetylates cohesin, we performed an experiment to separate DNA synthesis from cohesin acetylation. We utilized primer extension using Pol ε, which does not perform strand displacement synthesis. To ensure that DNA synthesis was complete, we incubated the reaction for 15 min. In one sample, cohesin and its cofactors were included during this incubation, whereas in another sample, cohesin was added only after the 15-min incubation. In that case, the incubation was extended for further 15 min (Figure 3B). As controls, cohesin was added to a continuous 30-min incubation or was added to a 30-min reaction with T7 DNA polymerase. As expected, cohesin acetylation was detected during the 30-min incubation with Pol ε, but not with T7 polymerase. Acetylation was also detected during the first 15-min replication interval, but strikingly, cohesin acetylation was more efficient during the second 15 min, during which no more DNA synthesis took place. This observation suggests that cohesin acetylation is not directly coupled to DNA synthesis. Rather, a product that forms by DNA synthesis, and then persists, appears to stimulate the acetylation reaction.

### PCNA on replicated DNA promotes cohesin acetylation

**Figure 4:**
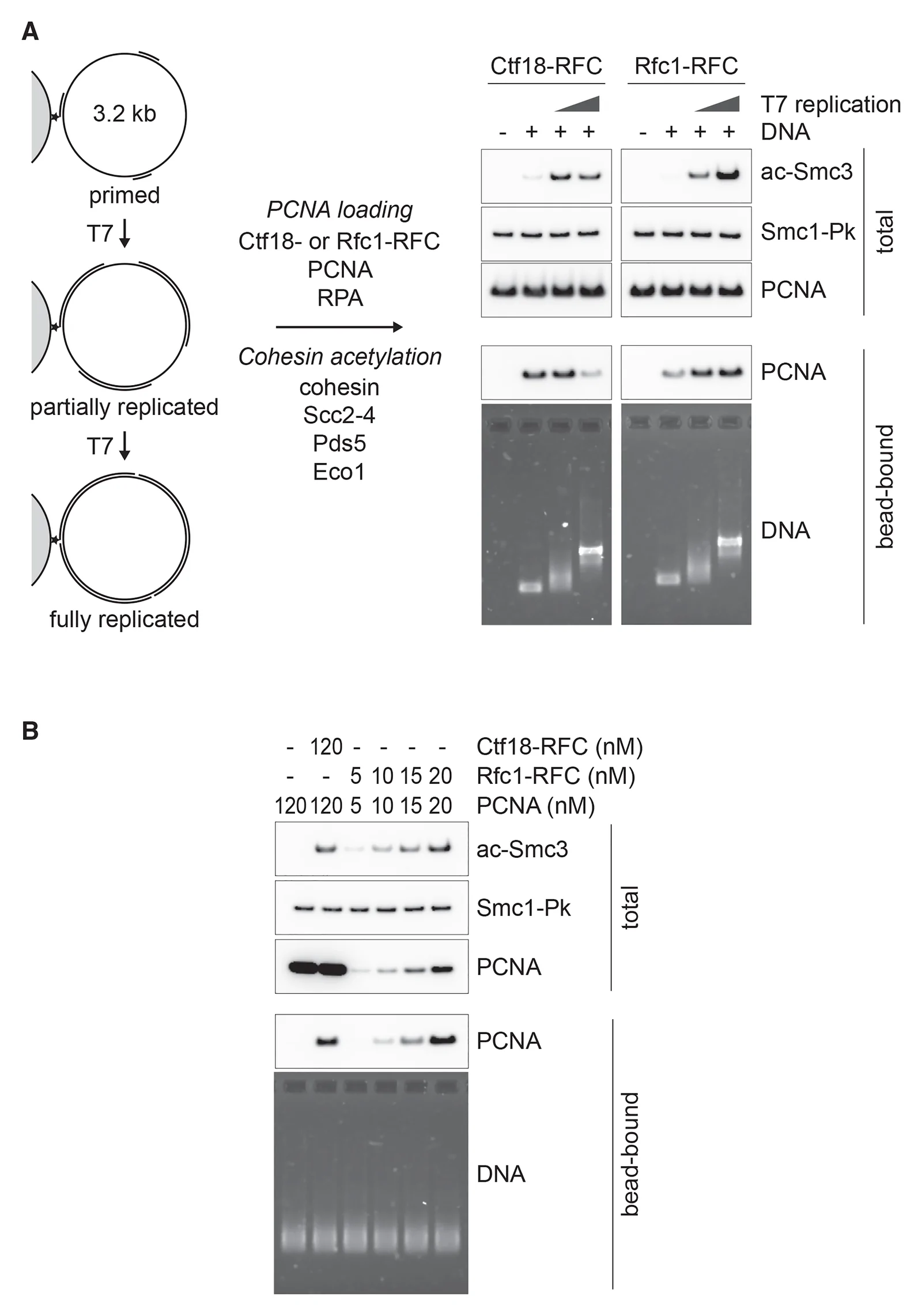
PCNA on fully replicated DNA stimulates cohesin acetylation (A) Partially or fully replicated DNA structures, generated by T7 DNA polymerase, served as substrate for PCNA loading by 20 nM Rfc1-RFC or 120 nM Ctf18-RFC. Cohesin and cohesion establishment factors were also added. A gel image of the immobilized DNA substrates is shown, together with immunoblot analyses of loaded PCNA and of cohesin acetylation. (B) Rfc1-RFC and Ctf18-RFC promote cohesin acetylation with equal efficiency. As (A), but the indicated concentrations of Ctf18-RFC, Rfc1-RFC, and PCNA were used for PCNA loading onto an immobilized, partially replicated DNA substrate. See also Figure S4, demonstrating that cohesin acetylation remains PCNA and Eco1 PIP box dependent when using premade replication intermediates.

Having established that cohesin acetylation does not require ongoing DNA synthesis, we prepared a series of DNA substrates that mimic replication products and investigated their ability to support cohesin acetylation. We annealed three primers to our circular ssDNA substrate and extended these either partially, or fully, using T7 DNA polymerase. DNA synthesis by this polymerase did not support cohesin acetylation above—here we merely utilize the generated DNA products. These products were purified and bound to streptavidin coated beads via a 5′ biotin moiety on one of the three primers (Figure 4A). Onto these substrates, PCNA was then loaded using either Ctf18-RFC, known to be required for *in vivo* cohesin acetylation,^44,45^ or Rfc1-RFC (Figure S4A). Cohesin and its cofactors were added and Smc3 acetylation assessed by immunoblotting. Bead-bound fractions were separately analyzed to monitor PCNA loading.

Consistent with previous reports,^46,47^ Ctf18-RFC efficiently loaded PCNA onto primed or partially replicated DNA but less so onto fully replicated DNA. Following addition of cohesin and its cofactors, almost no acetylation was detected with the primed substrate, while substantial acetylation was seen with both the partially and fully replicated DNAs (Figure 4A). Since only little PCNA was loaded onto the fully replicated circles by Ctf18-RFC, these observations suggest that PCNA on a fully replicated template is especially efficient at promoting cohesin acetylation, compared to PCNA on partially replicated DNA. Cohesin acetylation in this replication-independent setting remained reliant on the presence of PCNA and the Eco1 PIP box (Figure S4B).

We reached a similar conclusion when we used Rfc1-RFC to load PCNA onto the same three DNAs. As expected,^46,47^ Rfc1-RFC loaded PCNA with equal efficiency onto both the partially and the completely replicated templates. Compared with the partially replicated DNA, cohesin acetylation was substantially increased by the fully replicated DNA. We will further analyze the nature of the “fully replicated” structure below.

*In vivo*, Ctf18-RFC makes a key contribution to cohesin acetylation.^28^ However, both Ctf18 and Rfc1-RFC supported *in vitro* cohesin acetylation. To compare the proficiency of the two PCNA loaders side-by-side, we used the partially replicated DNA template, a preferred substrate for both RFC complexes. Higher Ctf18-RFC concentrations, compared with Rfc1-RFC, were required to achieve comparable PCNA loading, probably because the former requires its binding partner Pol ε for full activity.^48^ Nonetheless, similar amounts of loaded PCNA resulted in similar levels of cohesin acetylation (Figure 4B). Thus, it is the product of the PCNA loading reaction, not the identity of the loader, that promotes cohesin acetylation. The special Ctf18-RFC role during *in vivo* cohesion establishment remains to be fully understood.

### A nick or flap promote cohesin acetylation

**Figure 5:**
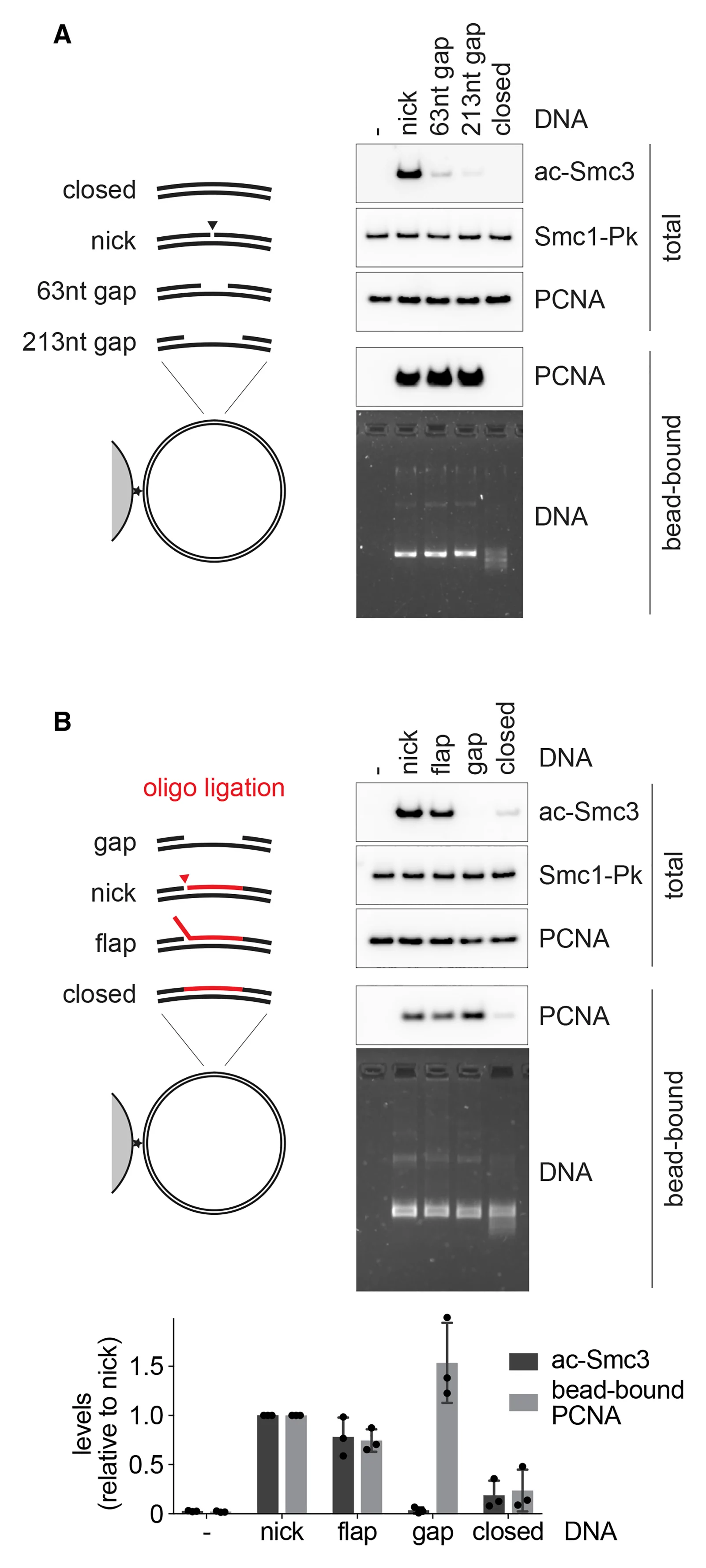
DNA nicks or flaps promote cohesin acetylation (A) Nicked, but not gapped, DNA stimulates cohesin acetylation. Schematic of the immobilized DNA substrates used, as well as gel image of these substrates following the PCNA loading and cohesin acetylation reaction. The bead-bound fractions were analyzed next to the total protein fractions for PCNA loading and Smc3 acetylation. See also Figure S5A, showing that single-stranded DNA, exposed in gapped DNA, does not inhibit cohesin acetylation. (B) As (A), but the 63 nt gap substrate was further modified by oligo ligation to recreate a nick, create a flap, or covalently close the DNA. PCNA loading and Smc3 acetylation were quantified in three independent repeats of the experiment and are plotted relative to the levels observed using the nicked DNA substrate. All experimental results are shown, the bar and whiskers represent mean and standard deviation. See also Figure S5B, confirming oligo ligation.

To understand which aspect of fully replicated DNA promotes cohesin acetylation, we prepared an additional series of defined DNA structures. We started from a synthetic, covalently closed, double-stranded DNA circle in which we had included an internally biotinylated primer. A nicking enzyme was used to introduce either a single nick or multiple adjacent nicks that allowed us to generate gaps of 63 or 213 nucleotides (nt) in lengths (Figure 5A). These substrates, reflecting concluding stages of DNA replication, were immobilized on beads, before we used Rfc1-RFC to load PCNA. Equal PCNA levels were loaded onto the nicked and gapped substrates, whereas no PCNA could be loaded onto the covalently closed circle. Upon addition of cohesin and cofactors, we observed efficient acetylation with the nicked, but hardly any acetylation using the 63 or 213 nt gapped DNA substrates. This suggests that a DNA nick preferentially activates cohesin acetylation.

An alternative explanation for the above result is that any 3′ end promotes acetylation, but that ssDNA exposed on gapped substrates has an inhibitory effect. To test this possibility, we used the nicked substrate but added a single-stranded oligonucleotide of the same length and sequence as the 63-nt gap. Even a large excess of this ssDNA did not inhibit cohesin acetylation (Figure S5A). Therefore, a nick is the required DNA substrate that stimulates cohesin acetylation in this setting.

Nicks arise as a transient intermediate during lagging strand DNA synthesis. As DNA Pol δ completes Okazaki fragment replication, strand displacement synthesis initially dislodges the RNA-DNA primer synthesized by Pol α-primase in the form of a flap. The flap is trimmed by the flap endonuclease Fen1 to result in a nick. We therefore wondered whether a flap structure also promotes cohesin acetylation. We utilized the 63-nt gap substrate to which we annealed and ligated oligonucleotides that either formed a 10-nt flap, recreated a nick, or covalently sealed the gap (Figures 5B and S5B). These substrates were immobilized, PCNA was loaded and cohesin acetylation assessed.

This experiment revealed that both nick and flap structures promote cohesin acetylation. Quantification of the acetylation signal, relative to the amount of loaded PCNA, showed that a nick or flap promote cohesin acetylation to an equal extent. Thus, two transient DNA structures that form during Okazaki fragment processing, a flap and a nick, promote cohesin acetylation.

### Okazaki fragment maturation by ligation terminates cohesin acetylation

Okazaki fragment maturation concludes when nicks are sealed by DNA ligase, resulting in covalently closed products. Covalently closed DNA substrates that were included in our previous reactions did not allow for a direct comparison with flapped or nicked DNAs, as RFC cannot load PCNA onto closed DNA circles. To address whether PCNA on covalently closed DNA supports cohesin acetylation, we therefore returned to replication-coupled cohesin acetylation reactions, to which we now added the budding yeast flap endonuclease Fen1 and DNA ligase Cdc9 (Figure S6).^41^ We used single-stranded circular DNA with three annealed primers as the substrate for Pol δ-catalyzed primer elongation reactions.

**Figure 6:**
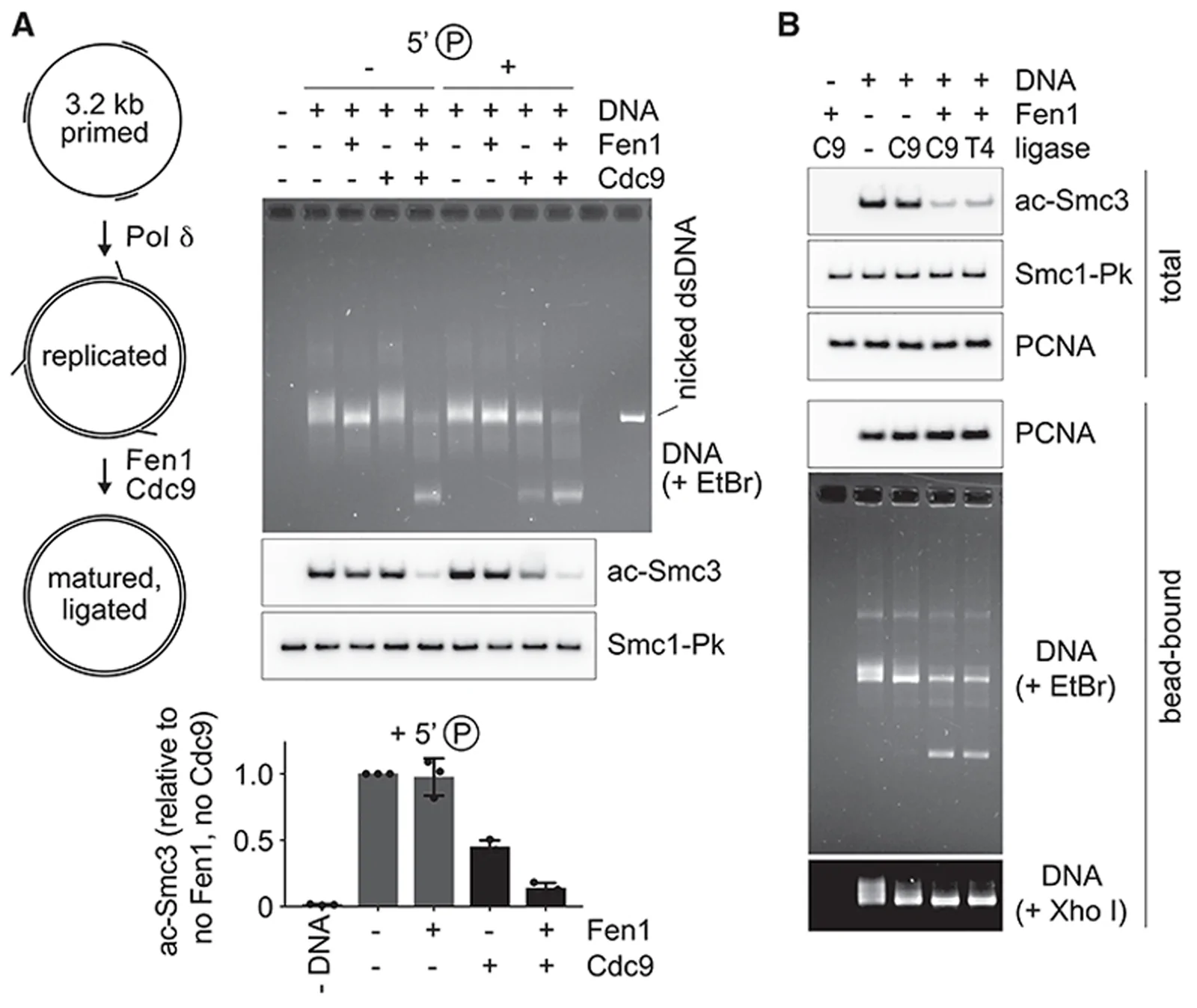
Nick ligation terminates cohesin acetylation (A) Replication product ligation terminates cohesin acetylation. Schematic of primed ssDNA replication by Pol δ in the absence or presence of Fen1 flap endonuclease and Cdc9 ligase. Cohesin and cohesion establishment factors were present in all cases, except Eco1, and incubated for 15 min. At this time, replication products in a reaction aliquot were analyzed by agarose gel electrophoresis in the presence of ethidium bromide (EtBr). Eco1 was added for an additional 20 min, and Smc3 acetylation was assessed by immunoblotting. The three oligonucleotide primers did, or not, contain a 5′ phosphate that allows direct ligation by Cdc9. Smc3 acetylation was quantified in 3 independent repeats of the experiment and is plotted relative to levels observed in the absence of Fen1 and Cdc9. All experimental results are shown, the bar and whiskers represent mean and standard deviation. (B) PCNA is retained on replication products following ligation. As (A), but an internally bio-tinylated primer was included to immobilize the DNA substrate. The bead-bound fractions were analyzed by immunoblotting next to the total protein fractions for PCNA loading and Smc3 acetylation. Replication products were analyzed by gel electrophoresis or alternatively were linearized by XhoI restriction before analysis. See also Figure S6 for SDS-PAGE analysis of purified Fen1 and Cdc9.

After incubation for 15 min without Fen1 or Cdc9, the replication products migrated slightly upward of the position of a double-stranded circular product, indicative of flaps produced by strand displacement synthesis. Addition of cohesin and its co-factors confirmed that these products are proficient in promoting cohesin acetylation (Figure 6A). Inclusion of Fen1 in the reaction resulted in replication products that were now of the expected double-stranded circular DNA size, suggesting successfulflap processing. As expected, the resultant nicked product also supported cohesin acetylation. Addition of both Fen1 and Cdc9 resulted in further marked changes. A substantial fraction of the replication products showed faster gel migration in the presence of ethidium bromide, a DNA intercalator that supercoils and thereby increases the mobility only of covalently closed circular products, confirming DNA ligation. Notably, ligation was accompanied by loss of cohesin acetylation, suggesting that nick ligation terminates the cohesin acetylation reaction.

Like Eco1, both Fen1 and Cdc9 interact with PCNA via their own PIP boxes. Competition for PCNA binding, rather than nick ligation, might therefore be a reason for reduced cohesin acetylation. To address this possibility, we performed a reaction without Fen1, in which the three oligonucleotide primers contained a 5′ phosphate that allows direct ligation (Figure 6A). Although less efficient than in presence of Fen1, ligation by Cdc9 reduced cohesin acetylation (note that three primers are present on our template and that ligation at all three junctions is required to observe a covalently closed product). Thus, ligation in the absence of Fen1 also stops cohesin acetylation.

To test whether Cdc9 competition for PCNA played a part in reduced cohesin acetylation, we replaced Cdc9 with T4 phage DNA ligase that does not contain a PIP box. In the presence of Fen1, both Cdc9 or T4 DNA ligase produced a similar amount of covalently closed product, and both resulted in a similar reduction of cohesin acetylation (Figure 6B). This suggests that conversion of a flapped or nicked DNA to a covalently closed replication product terminates the cohesin acetylation reaction. A reason for why ligation terminates cohesin acetylation could lie in reduced PCNA stability on DNA, following completion of replication maturation. To address this possibility, one of the primers in our last experiment contained an internal biotin moiety with which we immobilized the DNA substrate. This allowed us to monitor DNA-bound PCNA. Equal PCNA levels were retained on DNA, irrespective of the inclusion of Fen1 and/or DNA ligase in the reaction (Figure 6B). Therefore, PCNA that persists on DNA following ligation is no longer able to promote cohesion acetylation.

### The cohesin loader and Pds5 contribute to efficient cohesin acetylation

Above, we have identified transient DNA structures that promote cohesin acetylation. Previous studies have shown that on the cohesin side, all four subunits Smc1, Smc3, Scc1, and Scc3, as well as ATP hydrolysis, are required for efficient *in vitro* acetylation.^49,50^ *In vivo* observations have ascribed additional roles to cohesin’s Pds5 subunit, a putative Eco1 interactor,^51–53^ as well as to the Scc2-Scc4 cohesin loader.^12^ Whether the latter is required because cohesin must be loaded onto DNA for acetylation is not yet known. We therefore surveyed cohesin requirements in our replication-coupled acetylation assay.

**Figure 7:**
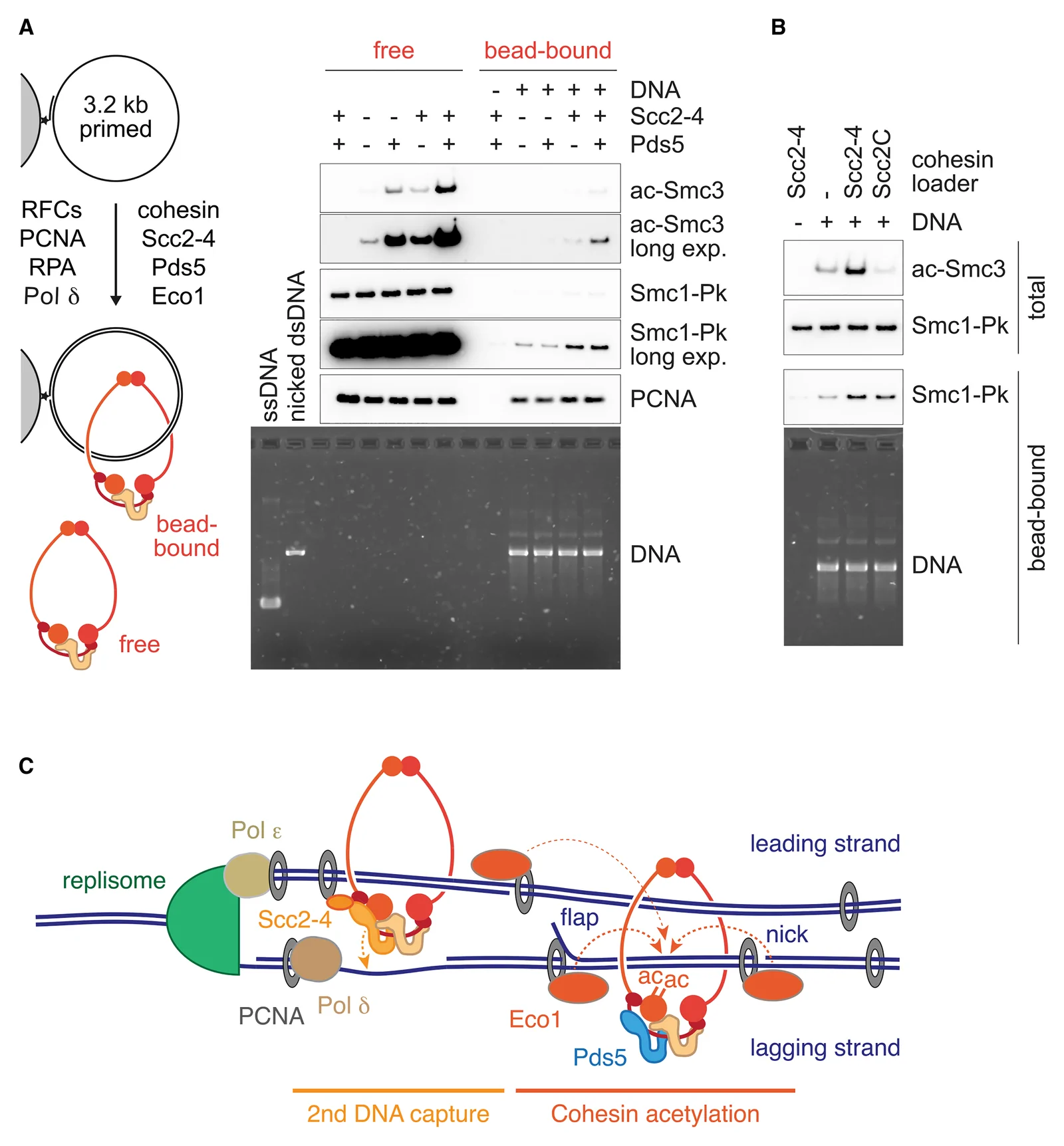
Scc2, Scc4, and Pds5 together promote Smc3 acetylation (A) Both DNA-bound and free cohesin becomes acetylated, dependent on Scc2-Scc4 and Pds5. Schematic of the replication-coupled cohesin acetylation reaction using a bead-bound, primed ssDNA substrate. Gel image and immunoblot analyses of the free and bead-bound fractions are shown. See also Figure S7A for an acetylation time course analysis of bead-bound and free cohesin, Figure S7B for confirmation that cohesin must be ATP hydrolysis proficient to serve as substrate and Figure S7C showing that cohesin acetylation only occurs on DNA. (B) Scc4 is required for efficient cohesin acetylation. As (A), but in presence of the indicated cohesin loader modules. See also Figure S7D, demonstrating a direct protein interaction between the cohesin loader and PCNA. (C) A model for sister chromatid cohesion establishment at the DNA replication fork. The double-stranded leading strand product, adjacent to single-stranded lagging strand DNA, are substrates for sequential cohesin loading (2nd DNA capture).^9^ Neighboring flap and nick structures that characterize Okazaki fragment maturation promote cohesin acetylation to stabilize newly established sister chromatid cohesion (see discussion for details).

We began by testing the contribution of the Scc2-Scc4 cohesin loader and of Pds5. Efficient acetylation was observed only in their presence. Omitting either one reduced acetylation, while acetylation was barely detected in the absence of both (Figure 7A). The additive effects of Scc2-Scc4 and Pds5 was surprising, since they compete for cohesin binding and likely obstruct access to the Smc3 acetylation sites.^20,22,54^ It could be that mutual competition between the cohesin loader and Pds5 leads to subunit exchange, possibly aided by ATP hydrolysis, during which the Smc3 lysines become accessible for acetylation.

When we separated DNA-bound and soluble fractions following the incubation, we detected the majority of acetylated cohesin in the soluble fraction (Figure 7A). Only a small amount of cohesin was recovered on DNA and was also acetylated. Given the requirement of the cohesin loader might cohesin be transiently loaded onto DNA for acetylation, before being released again into the supernatant? We consider this scenario unlikely for two reasons. First, acetylation stabilizes cohesin on DNA and impedes unloading.^20^ Second, time course analysis showed that acetylation increases simultaneously in both the soluble and DNA-bound cohesin pools (Figure S7A). Yet, as previously reported,^49,50^ an active cohesin ATPase was also required for acetylation (Figure S7B). A possible interpretation of these observations is that cohesin acetylation occurs during resolution of an ATP-bound cohesin loading intermediate, e.g., the DNA gripping state,^23^ but that resolution of this state not always result in productive cohesin loading.

As an additional test to ask whether cohesin acetylation occurs on DNA, we performed a primer elongation reaction in the presence of Eco1 but then added benzonase to degrade DNA before cohesin was added. If Eco1 is activated by replicated DNA and gains the ability to acetylate soluble cohesin, then DNA would no longer be required at the time of acetylation. However, this was not the case. DNA digestion abolished cohesin acetylation (Figure S7C). This outcome is consistent with a scenario in which cohesin acetylation occurs on DNA, during an intermediate step of cohesin’s ATP binding and hydrolysis cycle.

### Scc4 promotes cohesin acetylation and engages PCNA as a cohesin loader receptor

The Scc2-Scc4 cohesin loader consists of two modules. Scc4, bound to the Scc2 N terminus (Scc4-Scc2N), serves as a chromatin adaptor for the cohesin loader.^55–58^ The Scc2 C-terminal domain (Scc2C) in turn contains all so far known biochemical activities that catalyze cohesin loading.^21,42^ To our surprise, while Scc2C was proficient in loading cohesin onto DNA in a replication-coupled assay, cohesin acetylation was severely compromised without Scc4 (Figure 7B). This observation suggests that Scc4 takes part in the cohesin acetylation reaction, possibly by serving as an adaptor that directs the Scc2-Scc4 complex to a replication fork receptor. Among the proteins present in our replication-coupled cohesin acetylation assay, we found that the Scc2-Scc4 complex exhibits a direct physical interaction with PCNA (Figure S7D). This interaction was reduced when we tested either the Scc2C or Scc4-Scc2N modules in isolation, suggesting that the cohesin loader uses a multipronged interaction to associate with PCNA. This finding opens the possibility that PCNA supports cohesion establishment not only as receptor of the Eco1 cohesin acetyltransferase but also of the Scc2-Scc4 cohesin loader.

## Discussion

Replication-coupled cohesin acetylation, which stabilizes cohesin’s sister chromatid embrace, is a hallmark feature of eukaryotic sister chromatid cohesion establishment. Here, we developed a biochemical assay to investigate replication-coupled cohesin acetylation. This approach identified DNA structures that transiently form during Okazaki fragment maturation—flaps and nicks—as molecular determinants that control cohesin acetylation. Knowing the nature of the acetylation signal allows us to build a model for the establishment of sister chromatid cohesion at DNA replication forks.

### A model for the establishment of sister chromatid cohesion

Sister chromatid cohesion establishment involves two steps. First, cohesin co-entraps the two replication products. Then, cohesin is acetylated to stabilize the cohesive embrace. Previous biochemical analyses have suggested a mechanism for the first step. Following topological loading onto dsDNA, cohesin can topologically entrap a second ssDNA.^9^ A characteristic of replication forks is the double-stranded leading strand product that lies next to a single-stranded region of the lagging strand (Figure 7C). Cohesin loading at replication forks has been experimentally observed, both in yeast and in human cells.^59,60^ Our tentative finding that PCNA acts as a cohesin loader receptor lends further support to the idea that the replication fork forms a unique substrate where cohesin is loaded not only onto one, but two DNAs.

If cohesin embraces both dsDNA and ssDNA, what might happen while Pol δ carries out ssDNA to dsDNA conversion? ssDNA-bound cohesin does not impede DNA synthesis, at least by a small prokaryotic DNA polymerase,^9^ while other DNA translocases smaller than Pol δ have been observed to push cohesin.^61^ If Pol δ also pushes cohesin during DNA synthesis, this will deliver cohesin exactly to those places where DNA structures form that activate acetylation (Figure 7C). In this way, sister chromatid co-entrapment has been temporally and spatially coupled with cohesin acetylation.

*In vivo* experiments in budding yeast revealed increased cohesin acetylation following ligase Cdc9 depletion, when nicks persist after DNA replication.^28^ Cdc9 depletion also results in residual PCNA on chromosomes, as nick ligation precedes net PCNA unloading by Elg1-RFC.^62^ Persisting nicks and PCNA not only augmented cohesin acetylation but also improved sister chromatid cohesion establishment when it was compromised by the absence of Ctf18-RFC.^28^ These observations add physiological relevance to the cohesin acetylation mechanism that we have biochemically described.

### Transient DNA structures that promote cohesin acetylation

How does a flap or nick promote cohesin acetylation? In the simplest scenario, Eco1 recognizes and becomes activated by PCNA next to a flap or nick. Common to a flap and nick is a disrupted phosphate backbone that causes a kink in the double helix. Eco1 possesses an essential zinc finger^43^ that could participate in DNA kink recognition. Precedence exists in the zinc fingers of the DNA damage sensor PARP-1 that recognize nicks by the deformability of DNA.^63^ Although the mechanism of Eco1 activation remains to be investigated, PCNA association places Eco1 at a prime position to engage with a flap or nick. At the same time, the Eco1-PCNA interaction highlights the unresolved question of how PCNA access is distributed among the many DNA modifying enzymes that all engage with PCNA.^34^ Our results suggest that the DNA substrate itself plays a role. A flap or nick, exposed as Pol δ completes DNA synthesis, changes the PCNA surrounding such that Eco1 might gain an affinity advantage.

Once activated, Eco1 acetylates both cohesin that is loaded onto DNA, as well as cohesin that eventually remains soluble. In contrast, *in vivo* acetylation is typically restricted to chromatin-bound cohesin. The reason might be that soluble acetylated cohesin becomes the target of deacetylases. Following HDAC8 depletion from human cells, cohesin acetylation indeed became detectable in the soluble fraction,^39^ consistent with the possibility that acetylation extends to both chromatin-bound and soluble cohesin.

If PCNA-bound Eco1 is activated as it encounters flaps or nicks during Okazaki fragment processing on the lagging strand, what is the role of the cohesion establishment factor Ctf18-RFC, which loads PCNA predominantly, although not exclusively,^28,64^ on the leading strand? There might be more than one answer to this question. On the one hand, flaps or nicks are also encountered by PCNA on the leading strand, e.g., as the consequence of ribonucleotide excision.^65^ A cohesin acetylation signal might therefore also emerge from the leading strand (Figure 7C). In addition, leading strand PCNA might serve as the cohesin loader receptor that promotes cohesin recruitment to the replication fork. Ctf18-RFC could thus promote replication-coupled cohesin loading, in addition to cohesin acetylation, a possibility that finds support from genetic analyses.^45,66^

### Limitations of the study

Eco1 binds to any DNA *in vitro*, and despite our efforts, we have been unable to detect increased affinity to nicked substrates. We were also unable to directly measure Eco1 catalytic activation upon encountering a nicked substrate. A reason is the complexity of Eco1’s physiological substrate, the multi-subunit cohesin complex that itself engages with DNA. We cannot therefore exclude the possibility that cohesin itself contributes to DNA structure recognition. The cohesin loader plays an important role in our cohesion establishment model but is in fact not strictly essential during cohesion establishment.^10^ This observation points to parallel cohesion establishment pathways. If the replisome can pass through cohesin rings, acetylation at any time before or during fork passage should lead to successful cohesion establishment. This scenario is apparent in human cells, where two Eco1 orthologs share roles in cohesion establishment.^32^ One, Esco1, acetylates cohesin already before DNA replication, dependent on Pds5 but independently of replisomes. The other, Esco2, shows interactions with both PCNA, as well as the minichromosome maintenance (MCM) replicative helicase. To what extent the MCM interaction also promotes cohesin acetylation, or protects Esco2 from ubiquitin-mediated proteolysis, remains to be fully understood.^33,67–69^ Future experiments will aim to biochemically recapitulate cohesin acetylation during co-entrapment of DNA replication products to decipher the relative contributions of parallel cohesion establishment pathways.

## Star+Methods

### Key Resources Table

**Table:** 

REAGENT or RESOURCE	SOURCE	IDENTIFIER
Antibodies		
Mouse monoclonal anti-V5(Pk)	Bio-Rad	Cat# MCA1360; RRID:AB_322378
Mouse monoclonal anti-acetylated Smc3	Gift from Shirahige Laboratory	N/A
Mouse monoclonal anti-PCNA (5E6/2)	Cell Services Science Technology Platform, The Francis Crick Institute	N/A
Mouse monoclonal anti-HA (12CA5)	Ibidem	N/A
Mouse monoclonal anti-HA (F-7)	Santa Cruz Biotechnology	sc-7392; RRID:AB_627809
Mouse monoclonal anti-myc (9E10)	Bio-Rad	Cat# MCA2200; RRID:AB_324359
Chemicals, peptides, and recombinant proteins		
Rabbit IgG-Agarose	Merck	Cat# A2909
TALON Metal Affinity Resin	Clontech Laboratories	Cat# 635502
HiTrap Heparin HP 1ml	Cytiva	Cat# 17-0407-01
HiTrap SP HP 1ml	Cytiva	Cat# 29-0513-24
Superose 6 Increase 10/300 GL	Cytiva	Cat# 29-0915-96
Superdex 200 Increase 10/300 GL	Cytiva	Cat# 28-9909-44
Superdex 75 10/300 GL	Cytiva	Cat# 17-5174-01
cOmplete, EDTA-free Protease Inhibitor Cocktail	Merck	Cat# 04693132001
Pefabloc SC	Roche	Cat# 11429876001
SYBR Gold nucleic acid gel stain	ThermoFisher	Cat#S11494
Acetyl coenzyme A lithium salt	Merck	Cat# A2181
Dynabeads M-280 Streptavidin	ThermoFisher	Cat# 11206D
MicroSpin S-400 HR Columns	Cytiva	Cat# 27514001
T7 DNA polymerase (unmodified)	NEB	Cat# M0274
T4 DNA ligase	Promega	Cat# M180B
Nb.BssSI	NEB	Cat# R0681
Nt.BbvCI	NEB	Cat# R0632
Exonuclease III	ThermoFisher	Cat# EN0191
T5 Exonuclease	NEB	Cat # M0663
ATP	ThermoFisher	Cat# R0441
TCEP	Fluorochem Limited	Cat# M02624
3-Indoleacetic acid (‘auxin’)	Merck	Cat # I2886
Proteinase K	ThermoFisher	Cat# EO0491
Experimental models: Organisms/strains		
All yeast strains used in this study are listed in Table S1.	N/A	N/A
Oligonucleotides		
All oligonucleotide sequences used in this study are listed in Table S2.	N/A	N/A
Recombinant DNA		
pBluescript harboring ARS1	On et al.^70^	N/A
pET14b-Eco1	Liu et al.^28^	N/A
pET14b-Eco1^-pip^	Liu et al.^28^	N/A
pET14b-Eco1^-zf^	This study	N/A

## Resource Availability

### Lead contact

Further information and requests for resources and reagents should be directed to and will be fulfilled by the lead contact, Frank Uhlmann (frank.uhlmann@crick.ac.uk).

### Materials availability

All unique reagents generated in this study will be made available upon reasonable request without restrictions.

## Experimental Model and Subject Details

### Yeast strains

Budding yeast *S. cerevisiae* strains used in this study are listed in Table S1. Cells were grown in yeast peptone (YP) medium containing 2% glucose (YPD), 2% raffinose, or 2% raffinose + 2% galactose as carbon source at 30 °C. Cells expressing Eco1 under control of the methionine repressible *MET3* promoter were grown in YNB or CSM medium lacking methionine. To initiate degradation of auxin-inducible degron (aid)-tagged Eco1 or Hos1,^40^ 500 mM indole-3-acetic acid (auxin) was added to the growth medium.

To assess complementation of Eco1 function by phosphorylation site mutant Eco1 variants, 50,000 cells and their 10-fold serial dilutions were spotted on a YNB plate lacking methionine, as well as a YPD plate with added auxin. Cells were grown for 5 and 3 days on the two types of plate, respectively.

For monitoring cohesin acetylation by the Eco1 phosphorylation site mutants, cells were grown in CSM medium with glucose, lacking methionine and synchronized in G1 by α factor addition. 30 minutes before release from G1 arrest, cells were filtered and transferred into YPD medium containing α factor and auxin to deplete endogenous Eco1. Following this treatment, cells were released to progress through the cell cycle in YPD containing 8 mg/mL nocodazole and auxin for two hours. G2/M arrested cells were harvested and processed for SDS-PAGE analysis and immunoblotting.

For the experiment to deplete Hos1, without or with Eco1 overexpression, cells were grown in YP medium containing 2% raffinose and synchronized in G1 by α factor addition. 30 minutes before release from G1 arrest, auxin and 2% galactose were added to the cultures as indicated. Cells were then filtered and released for synchronous cell cycle progression in YP medium containing raffinose, as well as auxin and galactose as indicated. α factor was re-added after 60 minutes to impose arrest in the following G1. Aliquots of cells were taken in 15-minute intervals and processed for flow cytometry of DNA content and for SDS-PAGE and immunoblotting.

## Method Details

### Protein expression and purification

Cohesin, the Scc2-Scc4 cohesin loader complex, its Scc4-Scc2N and Scc2C modules, RPA, RFC, PCNA, DNA polymerases d and ε, Fen1, and Cdc9 (budding yeast ligase 1) were purified as previously described.^41,42,56^

### Pds5

Budding yeast cells overexpressing Pds5 were grown in YP medium containing 2% raffinose as the carbon source to an optical density of 1.0 at 30 °C. 2% galactose was then added to the culture to induce protein expression, and cells were further grown for 4 hours. Cells were collected by centrifugation, washed with deionized water, and suspended in Pds5 buffer (50 mM HEPES-KOH pH 7.5, 20% glycerol, 0.5 mM TCEP) containing 300 mM NaCl, 0.5 mM Pefabloc, as well as cOmplete-EDTA (both Roche) protease inhibitor cocktail. The cell suspension was frozen in liquid nitrogen, then cells were broken in a cryogenic freezer mill. The cell powder was thawed on ice, and further Pds5 buffer containing 300 mM NaCl, and protease inhibitors was added. The lysate was clarified by centrifugation at 20,000 x g for 1 hour. The clarified lysate was transferred to pre-equilibrated IgG agarose beads and incubated for 3 hours. The resin was washed with Pds5 buffer containing 300 mM NaCl and then incubated in Pds5 buffer containing 300 mM NaCl, 10 mM MgCl_2_ and 1 mM ATP for 15 minutes. The resin was washed again with Pds5 buffer containing 300 mM NaCl and incubated overnight in the same buffer containing 10 mg/ml PreScission protease. The eluate was collected, and Pds5 dilution buffer (50 mM HEPES-KOH pH 7.5, 20% glycerol, 0.5 mM TCEP, 10 mM NaCl) was added to adjust the salt concentration to 150 mM NaCl. The diluted sample was loaded onto a HiTrap Heparin (Cytiva) column, equilibrated with Pds5 buffer containing 150 mM NaCl. The column was developed with a linear gradient from 150 mM to 1 M NaCl in Pds5 buffer. The peak fractions were pooled and loaded onto a Superdex 200 Increase (Cytiva) gel filtration column that was equilibrated and developed with Pds5 gel filtration buffer (20 mM Tris-HCl pH 7.5, 150 mM NaCl, 10% Glycerol, 0.5 mM TCEP). The peak fractions were concentrated by ultrafiltration.

### Ctf18-RFC

Budding yeast harboring the Ctf18, Rfc2, Rfc3, Rfc4, Rfc5, Dcc1 and Ctf8 expression constructs were grown in YP medium containing 2% raffinose as the carbon source to an optical density of 1.0 at 30 °C. 2% galactose was added to the culture and the cells were further grown for 2 hours. Cells were collected by centrifugation, washed with deionized water and suspended in Ctf18 buffer (25 mM HEPES-KOH pH 7.6, 10% glycerol, 1 mM EDTA, 0.02% NP-40, 1 mM DTT) containing 300 mM NaCl, 0.5 mM Pefabloc, as well as cOmplete (-EDTA) protease inhibitor cocktail. The cell suspension was frozen in liquid nitrogen, then cells were broken in a freezer mill. The cell powder was thawed on ice, and further Ctf18 buffer containing 300 mM NaCl and protease inhibitors was added. The lysates were clarified by centrifugation at 20,000 x g for 1 hour. The clarified lysate was transferred to pre-equilibrated IgG agarose beads and incubated for 2 hours to capture the protein A tag attached to Ctf18. The resin was washed with Ctf18 buffer containing 300 mM NaCl and then incubated in Ctf18 buffer containing 300 mM NaCl, 10 mM MgCl_2_ and 1 mM ATP for 15 minutes. The resin was washed again with Ctf18 buffer containing 300 mM NaCl and incubated overnight in the same buffer containing 10 mg/ml PreScission protease. The eluate was loaded onto a HiTrap SP HP (Cytiva) column equilibrated with Ctf18 buffer containing 300 mM NaCl. The column was developed with a linear gradient from 300 mM to 1 M NaCl in Ctf18 buffer. The peak fractions were pooled and loaded onto a Superose 6 (Cytiva) gel filtration column that was equilibrated and developed with Ctf18 buffer containing 400 mM NaCl. The peak fractions were concentrated by ultrafiltration.

### Rfc1-RFC

RFC that we generally use in DNA replication reactions was purified following overexpression of its five subunits in budding yeast.^41^ However, the purification protocol does not preclude copurification of endogenous Ctf18-RFC. To allow a strict comparison between Rfc1-RFC and Ctf18-RFC, we therefore purified Rfc1-RFC from a yeast strain in which the gene encoding Ctf18 had been deleted. The isolation of Ctf18-RFC in turn included an affinity purification step that specifically selects for Ctf18 as the large RFC subunit (see above).

### Eco1

His_6_-Eco1, His_6_-Eco1^-pip^ (containing Q18A and L21A) and His_6_-Eco1^-zf^ (containing C35A and C38A changes) were expressed in *E. coli* BL21(DE3) pLysS for 18 hours at 19 °C after induction with 0.5 mM IPTG as described.^28^ The cells were collected by centrifugation, suspended in Eco1 buffer (50 mM HEPES-KOH pH 7.5, 10% glycerol, 0.5 mM TCEP) containing 250 mM NaCl, 0.1% Triton X-100, 0.5 mM Pefabloc, as well as cOmplete (-EDTA) protease inhibitor cocktail and frozen in liquid nitrogen. The suspension was thawed, and 30 U/ml benzonase, 2.5 mg/ml RNase A and 40 mM imidazole were added. Cells were broken by sonication. The lysates were clarified by centrifugation at 20,000 x g for 30 minutes. The clarified lysate was transferred to pre-equilibrated TALON (Takara) metal affinity resin and incubated for 1.5 hours. The resin was washed with Eco1 buffer containing 250 mM NaCl and 50 mM imidazole, then incubated in Eco1 buffer containing 250 mM NaCl, 50 mM imidazole, 10 mM MgCl_2_ and 1 mM ATP for 15 minutes. The resin was washed again and then incubated in Eco1 buffer containing 250 mM potassium glutamate, 200 mM imidazole, 0.1 mM acetyl-CoA for elution. The eluate was loaded onto a Superdex 75 (Cytiva) gel filtration column that was equilibrated with Eco1 buffer containing 250 mM potassium glutamate and 0.1 mM acetyl-CoA. The peak fractions were concentrated by ultrafiltration.

## DNA substrates

### ssDNA and primer annealing

pBluescript harboring ARS1 (3.2kb)^70^ was prepared from *E. coli* using the QIAGEN Plasmid Mega Kit. The double-stranded plasmid DNA was treated with the nicking enzyme Nb.BssSI (New England Biolabs). The nicked DNA was further treated with Exonuclease III (ThermoFisher Scientific) to degrade the nicked strand. A 3-fold molar excess of oligonucleotide MM256 was annealed to the resultant circular single-stranded DNA by heating to 90 °C, followed by gradual cooling to room temperature. Surplus oligonucleotide was removed by gel filtration using Sephacryl S-400 medium (Cytiva).

### DNA immobilization and ssDNA-to-dsDNA conversion

For immobilization on beads, three approximately equidistant oligonucleotides MM256 and MM211, as well as 5’-biotinylated oligonucleotide Y767, were annealed to the single-stranded plasmid DNA. Biotinylated DNA was incubated with M-280 Streptavidin Dynabeads (ThermoFisher Scientific) in buffer A (5 mM Tris-HCl pH 7.5, 0.5 mM EDTA, 1 M NaCl, 0.01% NP-40) at 30 °C for 30 minutes. The DNA-bound beads were washed with buffer A and then with primer extension buffer (25 mM HEPES/KOH pH 7.5, 250 mM potassium glutamate, 10 mM magnesium acetate, 1 mM DTT, 0.01% NP-40) before use. To convert the ssDNA to dsDNA, DNA beads were suspended in T7 buffer (20 mM Tris-HCl pH 7.5, 10 mM MgCl_2_, 1 mM DTT, 0.02% NP-40, 100 μg/ml BSA and 80 μM each of four dNTPs). 0.25 U/μl T7 DNA polymerase (New England Biolabs) was added and incubated for 1 minute or for 7.5 minutes at 25 °C, resulting in partially or fully converted DNA, respectively. The DNA beads were now washed with buffer B (10 mM Tris-HCl pH 7.5, 10 mM EDTA, 1 M NaCl, 0.02% NP-40, 1 mM DTT), and again with primer extension buffer before use.

### Internally biotinylated circular dsDNA with a nick, gap or flap

To create a single site-specific nick, the CCTCAGC recognition sequence for the Nt.BbvCI nicking enzyme was inserted into the pBluescript multiple cloning site by In-Fusion cloning (Takara). To allow the creation of gaps, oligos CB630 and CB631 were annealed to create a 71 bp double-stranded fragment containing five Nt.BbvCI recognition sites with HindIII compatible overhangs for insertion into pBluescript. Clones with a single (five Nt.BbvCI sites) and with three tandem repeat insertions (15 Nt.BbvCI sites) were selected. The three plasmids were purified and converted to circular ssDNA using Nb.BssSI and Exonuclease III treatment, as described above. Three oligonucleotides (MM254A, CB296 and internally biotinylated long TH46) were annealed. Second strand DNA synthesis of the primed ssDNA, as well as ligation were performed in T7 buffer containing 0.125 U/ml T7 DNA polymerase, 0.0375 U/ml T4 DNA ligase (New England Biolabs) and 2.5 mM ATP for 90 minutes at 30 °C. The products were then treated with T5 exonuclease (New England Biolabs) to degrade all but the biotinylated, covalently closed circular dsDNA products.

To introduce a single nick, the modified pBluescript plasmid was now treated with Nt.BbvCI. To introduce a single-stranded gap, the insertion-carrying plasmid were multiply nicked by Nt.BbvCI and then heated to 80 °C for 5 minutes. Short oligonucleotides (MM314, MM315, MM316, MM317, MM318), complementary to the released ssDNA fragments, were included in approximately 100-fold excess to capture released fragments as the temperature returned to ambient. Finally, the gapped DNA was separated from the released fragments and from free oligonucleotides by gel filtration as before.

To prepare flapped DNA, oligonucleotide MM320 that contains a 10-nucleotide flap extension was annealed to the short-gapped DNA prepared above. Nicked DNA was prepared similarly, but oligonucleotide MM319 lacking the extension was used instead. Alternatively, MM319 that was phosphorylated at its 5’ end was used to allow for complete ligation to obtain covalently closed circular DNA. Following annealing, surplus oligonucleotides were removed by gel filtration and the annealed primers were ligated to template DNA using T4 ligase. The internally biotinylated DNAs were then immobilized on streptavidin beads as described above.

### Replication-coupled cohesin acetylation assay

In a standard reaction in solution, 4 nM ssDNA with annealed oligonucleotide MM256, 400 nM RPA, 70 nM PCNA, 60 nM RFC, 60 nM Ctf18-RFC, 150 nM cohesin, 150 nM Scc2-Scc4, 150 nM Pds5, 35 nM or 70 nM Eco1 were combined in primer extension buffer containing 5 mM ATP, 80 μM each of the four dNTPs and 0.5 mM acetyl-CoA. Then 20 nM Pol ε or 4 nM Pol δ, if not stated otherwise, were added to the mixture and incubated at 30 °C for 12 minutes, or longer if indicated. For protein analysis, an aliquot of the reaction was added to SDS sample buffer, and the mixture was boiled. The denatured proteins were separated using SDS-PAGE and transferred to a nitrocellulose membrane for immunoblotting. To analyze DNA, another aliquot of the reaction was transferred into deproteinization buffer (10 mM Tris-HCl pH 7.5, 1 mM EDTA, 50 mM NaCl, 0.75% SDS, 1 mg/ml protease K) and incubated at 50 °C for 20 minutes. The deproteinized DNAs were further purified using phenol-chloroform extraction before application to TAE agarose gel electrophoresis. The single-stranded plasmid DNA, next to nicked double-stranded DNA, prepared by treating covalently closed circular plasmid DNA with Nb.BssSI, were loaded for reference. DNA was stained using SYBR Gold, and gel images were captured using an Amersham Imager 600 or a GelDoc XR+ Documentation System (Bio-Rad).

To analyze proteins specifically associating with DNA replication products, the biotinylated-primed ssDNA substrate (using oligonucleotide Y767) was immobilized on streptavidin beads as described. DNA synthesis was carried out essentially under the same condition when compared to in-solution reactions, but the primer extension reactions proceeded for 20 minutes. DNA-bound magnetic beads were then collected using a magnetic stand and separated from the supernatant fractions. The beads were washed with primer extension buffer containing 0.1% NP40. To analyze DNA-bound proteins, SDS sample buffer was then added to the washed beads and boiled, before loading for SDS-PAGE analysis. To analyze DNA products, an aliquot of the DNA beads was incubated in deproteinization buffer for 20 minutes at 50 °C, and DNA products were retrieved and separated from the beads using phenol-chloroform extraction.

### Cohesin acetylation following direct PCNA loading onto immobilized DNA

Single-stranded plasmid DNA annealed to oligonucleotides MM256, MM211 and 5’-biotinylated Y767 was immobilized on streptavidin beads. Primers were not, partially, or fully elongated using T7 DNA polymerase as described. Then the DNA beads were suspended in primer extension buffer containing 100 nM RPA, 5-120 nM PCNA, 120 nM Ctf18-RFC, 5-20 nM Rfc1-RFC, 150 nM cohesin, 150 nM Scc2-Scc4, 150 nM Pds5, 35 nM Eco1, 5 mM ATP and 0.5 mM acetyl-CoA. The DNA beads were incubated at 30 °C for 20 minutes. DNA-bound proteins and the DNA were analyzed as described above. Alternatively, the various internally biotinylated circular plasmid DNAs with or without a nick, gap or flap, described above, were used as substrates in this cohesin acetylation reaction.

### Replication maturation and ligation assays

Replication was carried out using single-stranded plasmid DNA annealed to three oligonucleotide primers, MM254A, CB296 and CB408. In reactions with immobilized DNA, internally biotinylated long TH46 was used instead of CB408. Replication and cohesion factors were used as before, except 10 nM Pol δ, 35 nM Fen1 and 140 nM Cdc9 or 0.14 U/ml T4 DNA ligase were included as indicated.

## Quantification and Statistical Analysis

Conclusions in this study that are based on qualitative comparisons, where effect sizes were vast, are based on at least three independent experimental repeats. In these cases, representative experiments are shown. In cases where quantitative comparisons were warranted, again three independent repeats of each experiment were performed. Band intensities following immunoblotting and detection using chemiluminescence reagents (Millipore) were quantified with an Amersham Imager 600. Individual results from all three repeats are shown, together with the means and standard deviations.

## Supplementary Material

Supplemental information can be found online at https://doi.org/10.1016/j.cell.2022.12.044.

Document S2

Table S1

Supplemental figures

## Data Availability

All data reported in this paper will be shared by the lead contact upon request. This paper does not report original code. Any additional information required to reanalyze the data reported in this paper is available from the lead contact upon request.

## In brief

Minamino et al. uncover that transient DNA structures that form at replication forks serve as a molecular clue that couples DNA replication to sister chromatid cohesion establishment.

## Highlights

- Biochemical reconstitution of DNA-replication-coupled cohesin acetylation
- Flaps or nicks, transient DNA structures during replication, promote acetylation
- Acetylation occurs next to where cohesin likely co-entraps the replication products
- A model for replication-coupled sister chromatid cohesion establishment emerges
