# Supplementary material for: A replication fork determinant for the establishment of sister chromatid cohesion
Source: Cell. Author manuscript; Available in PMC 2026 Aug 28. (PMC7619413; doi:10.1016/j.cell.2022.12.044)
Supplement: Supplementary file 1 [file EMS217829-supplement-Document_S2.pdf]

Supplemental References

S1. Minamino, M., Higashi, T.L., Bouchoux, C., and Uhlmann, F. (2018). Topological in vitro loading of the budding yeast cohesin ring onto DNA. Life Sci. Alliance *1*, e201800143

S2. Posse, V., Johansson, E., and Diffley, J.F.X. (2021). Eukaryotic DNA replication with purified budding yeast proteins. Methods Enzymol. *661*, 1-33.
