# Supplementary material for: A replication fork determinant for the establishment of sister chromatid cohesion
Source: Cell. Author manuscript; Available in PMC 2026 Aug 28. (PMC7619413; doi:10.1016/j.cell.2022.12.044)
Supplement: Supplementary file 2 [file EMS217829-supplement-Supplemental_figures.pdf]

Supplemental figures

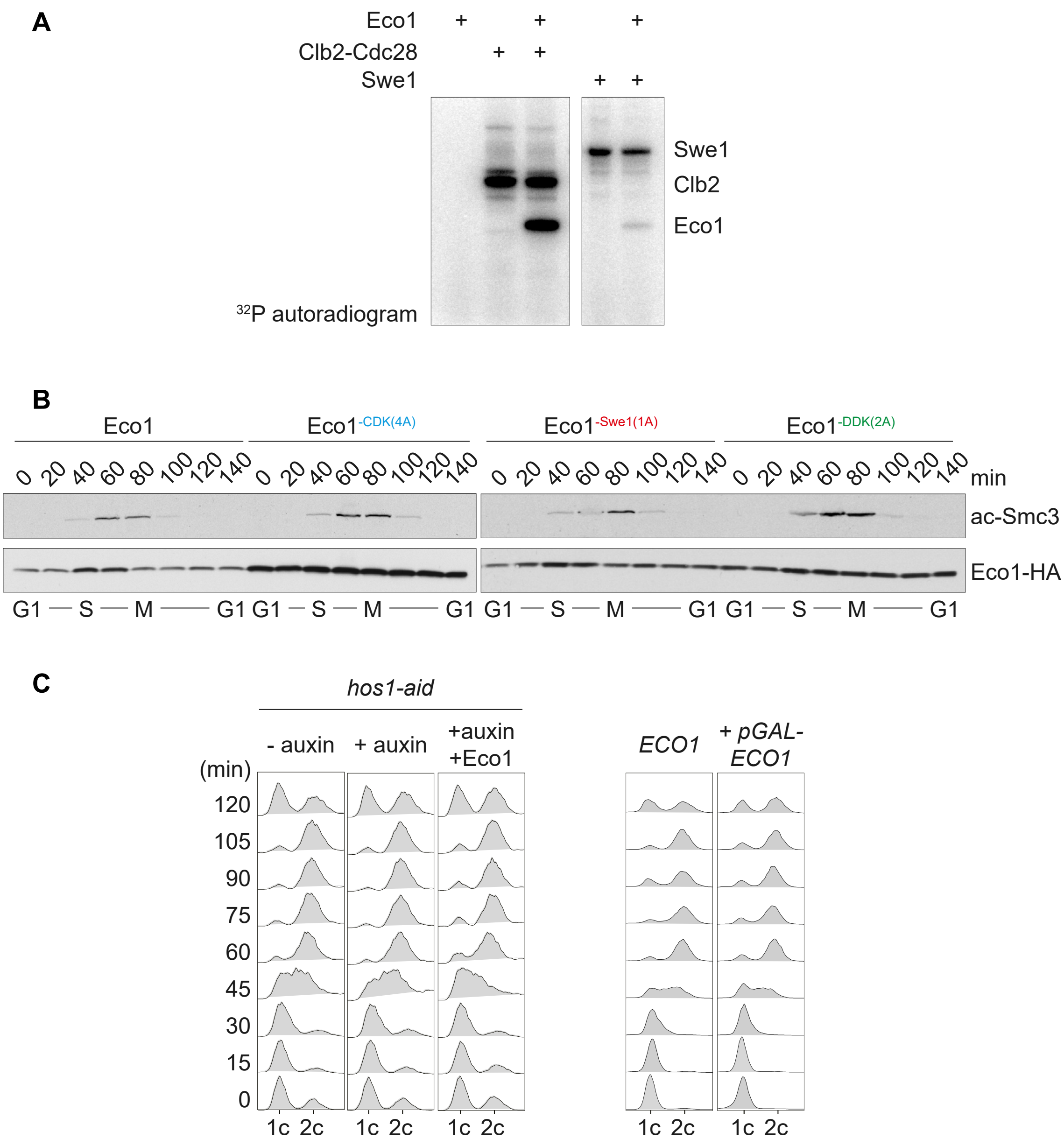

Figure S1. S phase-specificity of cohesin acetylation, related to Figure 1

(A) Confirmation of Eco1 phosphorylation by Swe1. The cyclin Clb2 or Swe1 kinase, fused to protein A tags, were overexpressed from multicopy plasmids under control of the *GAL1-10* promoter by addition of 2% galactose to YP medium containing 2% raffinose for 2 h. Cells were collected and lysed in 25 mM Tris-HCl pH 7.5, 250 mM NaCl, 0.1% Triton X-100, 1 mM $MgCl_2$, 0.5 mM TCEP, 10% glycerol, complete protease inhibitors, 2 U/μL benzonase. The Clb2/Cdc28 complex was purified as described[71] while Swe1 was pulled down on IgG-Dynabeads. Purified recombinant Eco1[28] was incubated with Clb2-Cdc28 or Swe1 in kinase buffer (50 mM Tris-HCl pH 7.5, 150 mM NaCl, 10 mM $MgCl_2$, 0.1 mM ATP, spiked with γ-^{32}P-ATP) for 30 min at 30°C. The reactions were terminated by addition of SDS loading buffer for analysis of phosphate incorporation by SDS-PAGE followed by autoradiography.

(B) Unaltered Smc3 acetylation timing in cells lacking Eco1 phosphorylation sites. Cells lacking both endogenous Eco1 and Wapl (*eco1Δ wpl1Δ*)[11] and ectopically expressing the indicated Eco1 variants were grown in YP medium containing 2% glucose and arrested in G1 by α factor addition. Cells were then washed and released (0 min) to progress synchronously through the cell cycle. α factor was re-added after 60 min to impose re-arrest in the following G1. Aliquots of cells were taken at the indicated times and processed for SDS-PAGE and immunoblotting. Cell cycle stages, based on flow cytometry analysis of DNA content, are indicated.

(C) Flow cytometric analyses of DNA content to confirm synchronous cell cycle progression of the cultures used for the experiment shown in Figure 1B.

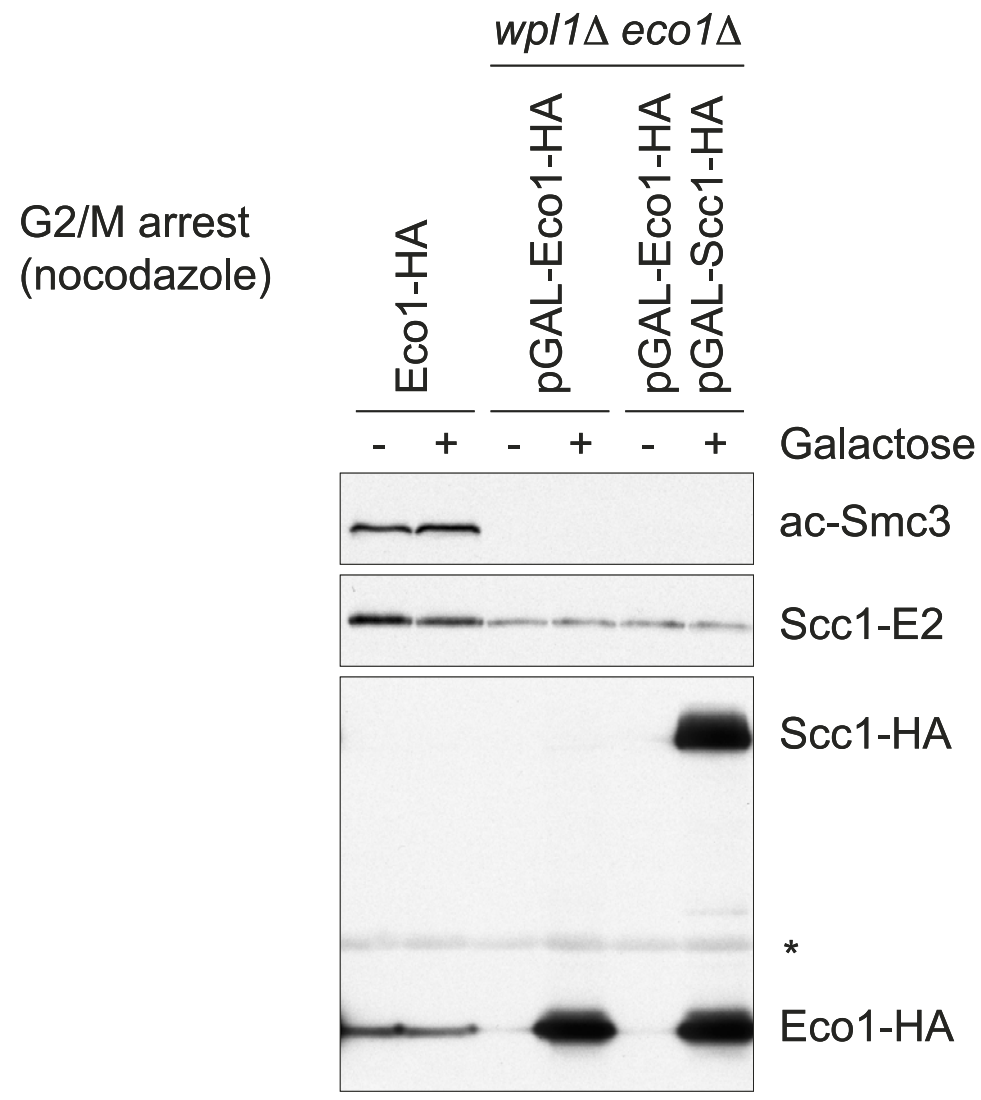

Figure S2. Smc3 acetylation is restricted to S phase, related to Figure 1

Cells containing wild-type endogenous Eco1 (fused to an HA epitope-tag for detection), or lacking Eco1 and kept viable by the absence of Wapl (*eco1Δ wpl1Δ*),[11] were grown in YP medium containing 2% raffinose, synchronized in G1 by α factor block and release, then arrested in G2/M in medium containing 8 μg/mL nocodazole for 2 h. 2% galactose was then added for an additional 1 h to induce expression of the indicated proteins under control of a galactose-inducible promoter (*pGAL*). Cells were harvested and processed for SDS-PAGE and immunoblotting. The control strain exemplifies the expected Smc3 acetylation levels following DNA replication. In contrast, G2/M induction of Eco1 (or of Eco1 together with Scc1 to afford additional cohesin loading onto chromosomes at this cell cycle stage)[10] did not result in detectable Smc3 acetylation.

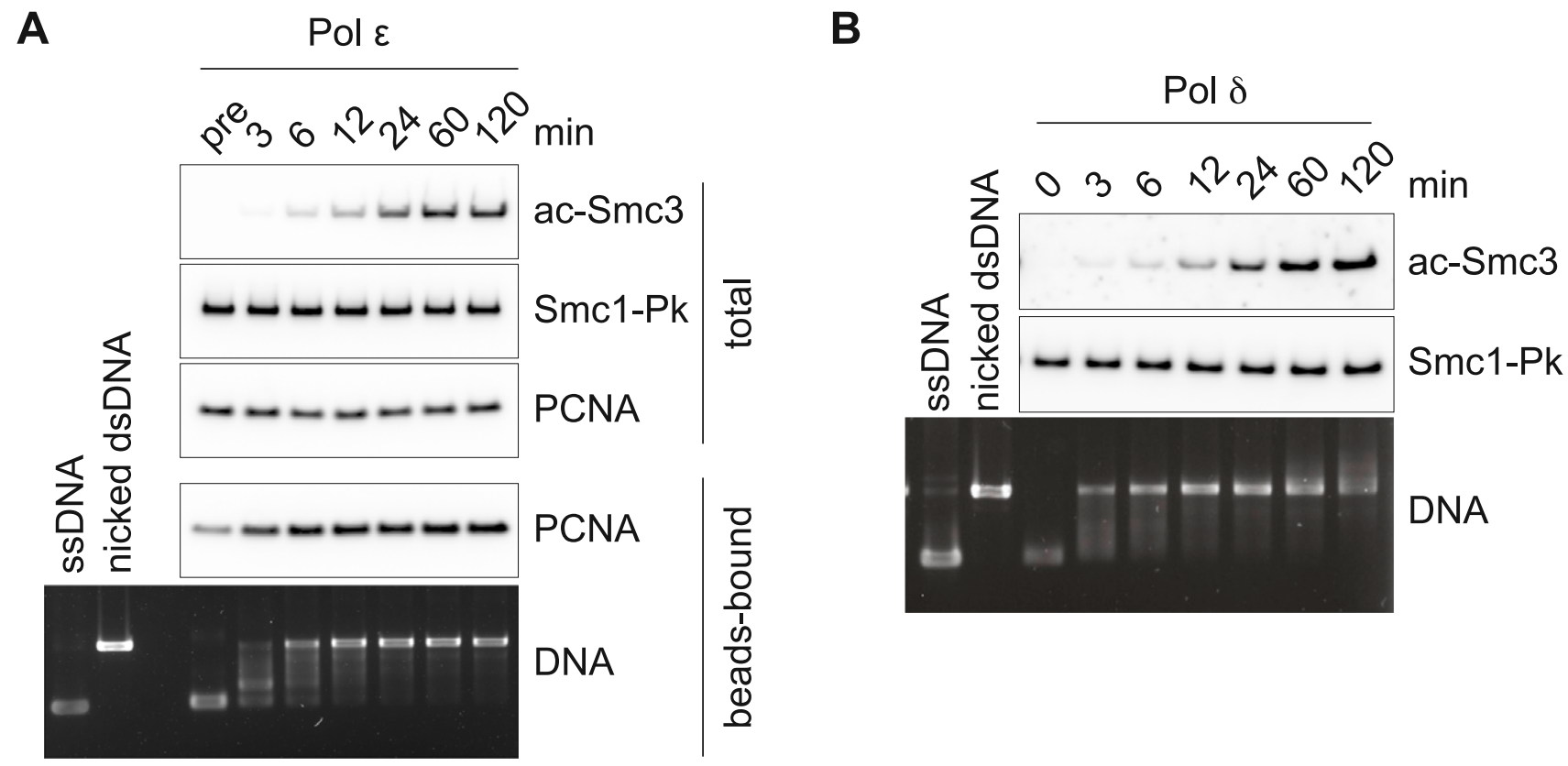

Figure S3. Cohesin acetylation following completion of DNA synthesis, related to Figure 3

(A) Time course analysis of replication-coupled cohesin acetylation in a reaction in which Pol ε extends a bead-bound, primed circular ssDNA substrate. Samples were taken at the indicated times. A gel image of the replication products is shown, as well as immunoblot analysis of Smc3 acetylation. A Pk epitope-tag on the cohesin subunit Smc1 served as a loading control. PCNA loading onto DNA was analyzed in parallel by comparing levels in the total and bead-bound fractions. The first time point at 0 min (“pre”) was taken after mixing all components, but before placing the reaction at its incubation temperature of 30°C. This analysis shows that Smc3 acetylation becomes detectable with a delay after PCNA loading, once the first DNAs are fully replicated at 6 min. Acetylation then continues to increase even following completion of DNA synthesis at 12 min.

(B) As (A) but using Pol δ for DNA synthesis using a soluble, primed circular ssDNA substrate. ssDNA to dsDNA conversion is largely complete after 6 min, while strand displacement synthesis becomes apparent at later time points.

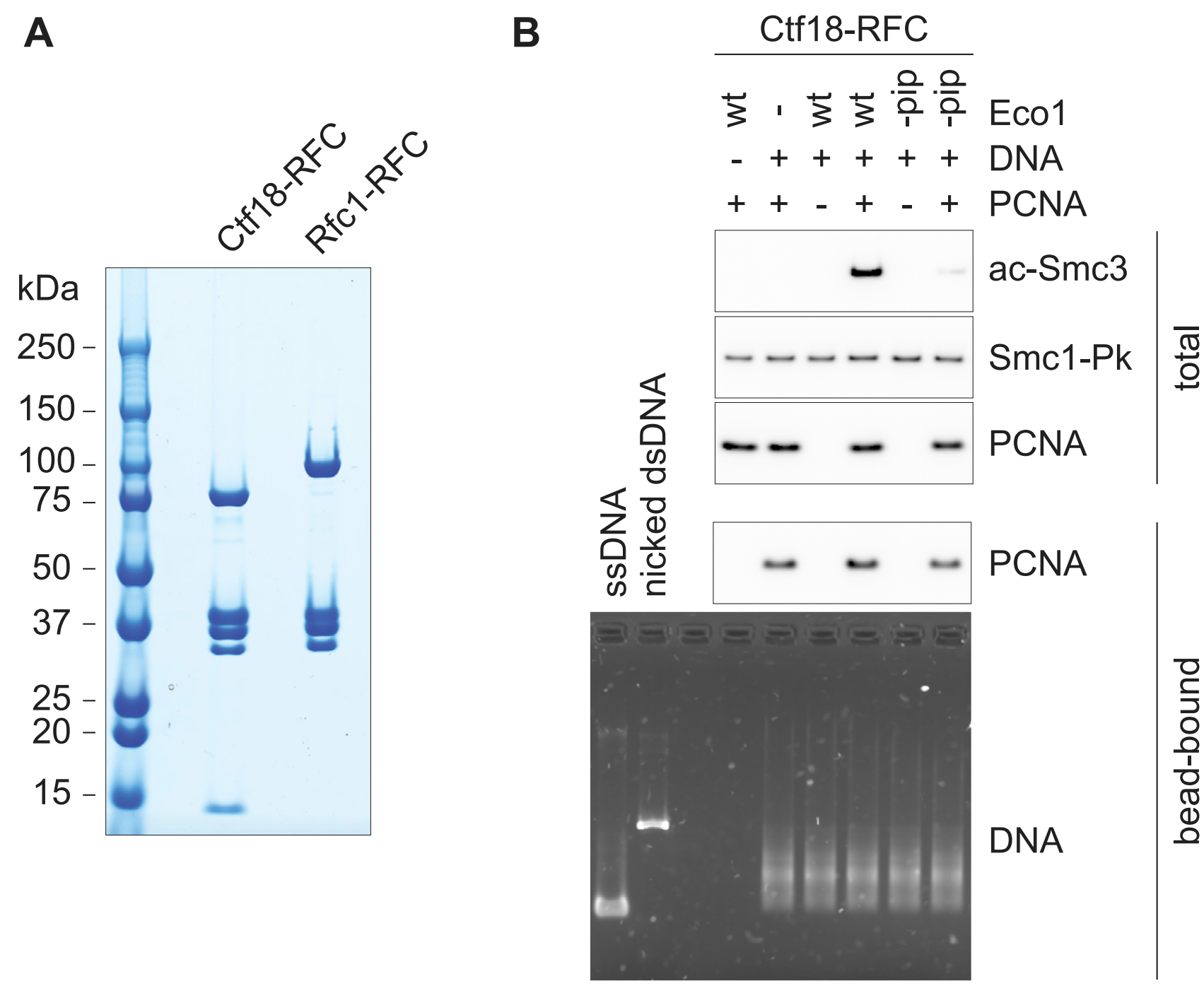

Figure S4. Cohesin acetylation remains PCNA and Eco1 PIP box-dependent when Ctf18-RFC loads PCNA onto a partially converted substrate, related to Figure 4

(A) Purified Ctf18-RFC and Rfc1-RFC were analyzed by SDS-PAGE and Coomassie blue staining.

(B) PCNA was loaded by Ctf18-RFC onto an immobilized, partially replicated DNA substrate as in Figure 4. Wild-type Eco1, or PIP box mutant Eco1^{-pip}, was included in the cohesin acetylation reaction. The dependence of cohesin acetylation on the Eco1-PCNA interaction was further confirmed by omission of PCNA from the reaction. A gel image of the immobilized DNA substrates is shown, together with immunoblot analyses of loaded PCNA, as well as of cohesin acetylation.

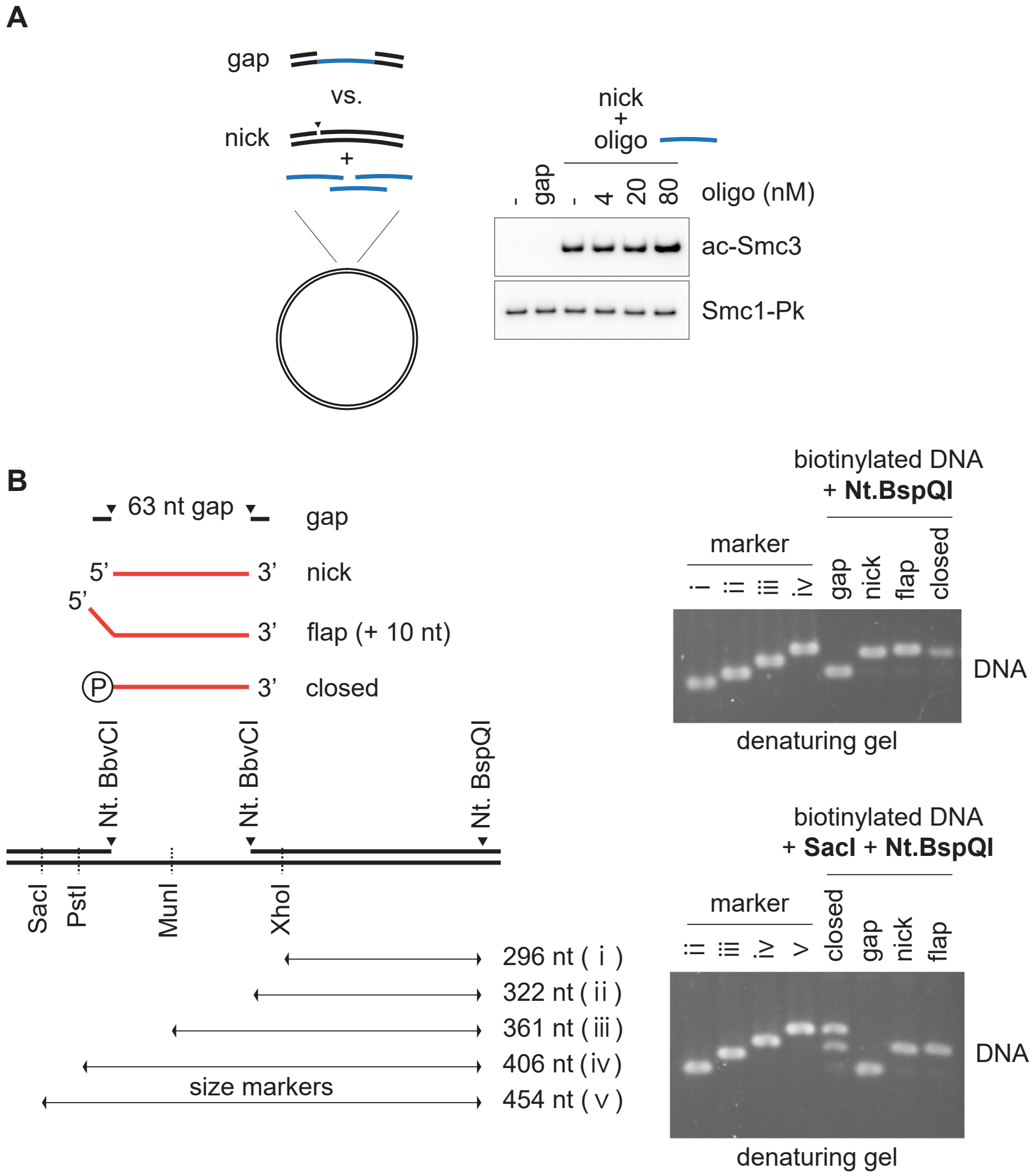

Figure S5. A DNA nick or flap promotes cohesin acetylation, related to Figure 5

(A) Single-stranded DNA, exposed in gapped DNA, is not inhibitory to cohesin acetylation. A nicked or gapped DNA substrate (4 nM), as in Figure 5, served as substrate for a PCNA loading and cohesin acetylation reaction. Oligonucleotides of the same length and sequence as that exposed on the gapped substrate were added at the indicated concentrations. The presence even of a high concentration of single-stranded oligonucleotide did not inhibit Smc3 acetylation.

(B) Schematic of the oligo ligation approach used in Figure 5B. Test digests were performed using nicking enzyme Nt.BspQI that once cleaves the strand to which the oligos were ligated. Digests were analyzed by denaturing 1 M urea/TBE gel electrophoresis. This revealed essentially complete conversion of the gapped input DNA to nicked or flapped products. Following gap closure using the 5′-phosphorylated oligo, no nicking product in the same size range should be detectable. However, a residual band of similar size to the nicked species suggests that gap closure at the 5′ end was incomplete. To visualize both nicked and closed molecules side by side, Nt.BspQI treatment was combined with restriction enzyme SacI digestion. This confirmed that the majority of products in the "gap closure" reaction were covalently closed. Size markers were generated from the original double-stranded plasmid DNA using Nt.BspQI and restriction enzymes as indicated.

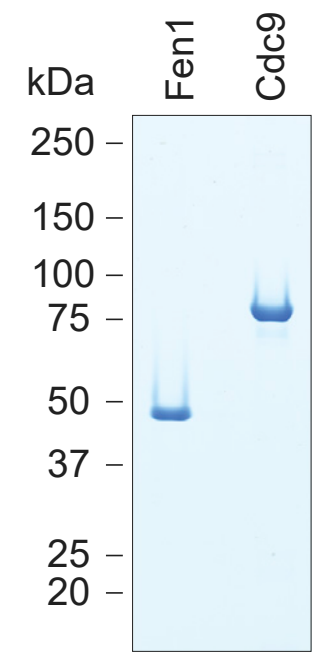

Figure S6. SDS-PAGE analysis of purified Fen1 and Cdc9, related to Figure 6
Purified Fen1 and Cdc9 were analyzed by SDS-PAGE followed by Coomassie blue staining. The position of size markers in indicated.

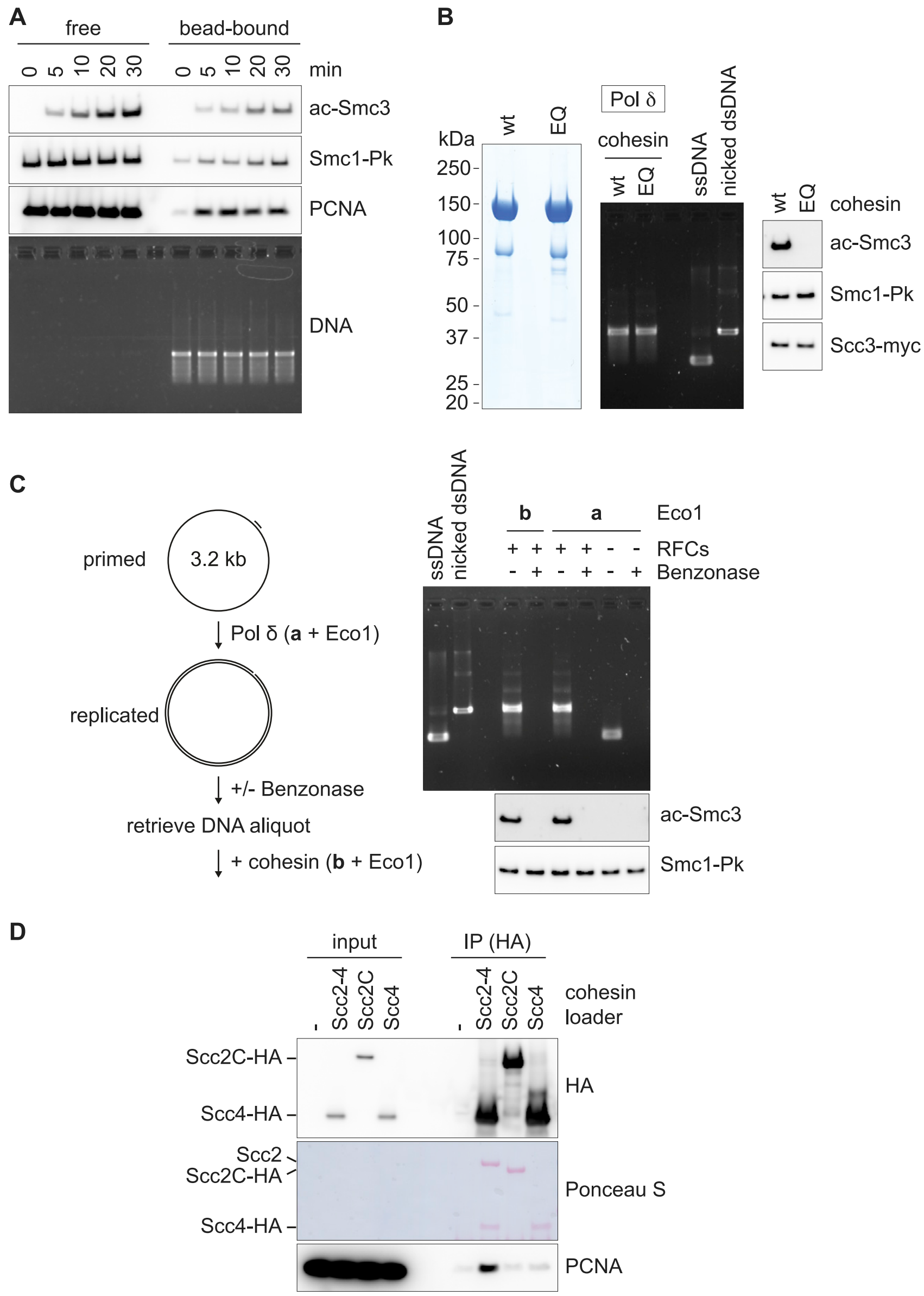

Figure S7. Cohesin substrate requirements for Smc3 acetylation, related to Figure 7

(A) Time course analysis of the free and bead-bound fractions for cohesin acetylation. A fully replicated, immobilized DNA substrate was prepared essentially as in Figure 4A, using T7 DNA polymerase and one biotinylated oligonucleotide primer. PCNA was then loaded in the presence of cohesin and its cofactors. Samples were taken at the indicated times and DNA and protein content of the free and bead-bound fractions were analyzed. Smc3 acetylation becomes simultaneously detectable in both the free and bead-bound fractions (note that cohesin loading onto DNA reached greater levels in this assay, when compared to the replication-coupled assay shown in Figure 7A, maybe due the presence of additional DNA-binding proteins in the latter).

(B) Cohesin must be ATP hydrolysis proficient to serve as acetylation substrate. Purified wild-type cohesin was analyzed next to Walker B motif mutant cohesin (Smc1 E1158Q, Smc3 E1155Q; EQ)[42] by SDS-PAGE followed by Coomassie staining. Either wild type or EQ cohesin was then included in a replication-coupled cohesin acetylation reaction. A gel image of the replication products is shown, as well as immunoblot analysis of Smc3 acetylation. Acetylation of wild-type cohesin, but not of EQ cohesin, was detected.

(C) Cohesin acetylation occurs on DNA. A singly primed ssDNA substrate was extended using Pol δ for 12 min. Products were then treated with (or without) benzonase for 3 min, and a sample taken for DNA analysis. Cohesin was then added for a 12-min acetylation incubation. Eco1 was present throughout (a) or was only added together with cohesin (b). Even when Eco1 was present throughout both incubations, its acetylation potential was abolished by benzonase treatment. This suggests that cohesin acetylation only occurs in the context of DNA.

(D) A direct protein interaction between the cohesin loader and PCNA. The Scc2-Scc4 cohesin loader complex (Scc2-4), or its Scc2C or Scc4-Scc2N ("Scc4") modules (100 nM each) were incubated with PCNA (200 nM). The respective cohesin loaders were then immunoprecipitated via an HA epitope tag. The input and precipitated fractions were analyzed by immunoblotting. PCNA coprecipitated with the Scc2-Scc4 complex, but not with Scc2C or Scc4, suggesting a multi-pronged cohesin loader interaction with PCNA that involves both Scc2C and Scc4 modules.
