# Supplementary material for: A replication fork determinant for the establishment of sister chromatid cohesion
Source: Cell. Author manuscript; Available in PMC 2026 Aug 28. (PMC7619413; doi:10.1016/j.cell.2022.12.044)
Supplement: Supplementary file 3 [file EMS217829-supplement-Table_S1.pdf]

Cell, Volume *186*

Supplemental information

A replication fork determinant

for the establishment of sister chromatid cohesion

Masashi Minamino, Céline Bouchoux, Berta Canal, John F.X. Diffley, and Frank Uhlmann

Table S1. Yeast Strains, Related to STAR Methods

The following budding yeast *S. cerevisiae* strains of the W303 background (an exception indicated) were used in this study.

Figure 1A and 1B

Y6034: *MAT* **a**, *pADH1-OsTIR1-myc*$_9$*::ADE2, SCC1-E2*$_6$*::K.l.TRP1, (HIS3MX6)pMET3-ECO1-IAA17::KAN*^{R}*MX6, pECO1-ECO1-HA*$_3$*::URA3*

Y6654: *MAT* **a**, *pADH1-OsTIR1-myc*$_9$*::ADE2, SCC1-E2*$_6$*::K.l.TRP1, (HIS3MX6)pMET3-ECO1-IAA17::KAN*^{R}*MX6, YIplac211::URA3*

Y6655: *MAT* **a**, *pADH1-OsTIR1-myc*$_9$*::ADE2, SCC1-E2*$_6$*::K.l.TRP1, (HIS3MX6)pMET3-ECO1-IAA17::KAN*^{R}*MX6, pECO1-ECO1*^{Y164A} *-HA*$_3$*::URA3* (Eco1$^{Swe1(1A)}$)

Y6656: *MAT* **a**, *pADH1-OsTIR1-myc*$_9$*::ADE2, SCC1-E2*$_6$*::K.l.TRP1, (HIS3MX6)pMET3-ECO1-IAA17::KAN*^{R}*MX6, pECO1-ECO1*^{S98A,S104A}*-HA*$_3$*::URA3* (Eco1$^{Ddk(2A)}$)

Y6657: *MAT* **a**, *pADH1-OsTIR1-myc*$_9$*::ADE2, SCC1-E2*$_6$*::K.l.TRP1, (HIS3MX6)pMET3-ECO1-IAA17::KAN*^{R}*MX6, pECO1-ECO1*^{S67A,T94A,S99AS,105A}*-HA*$_3$*::URA3* (Eco1$^{Cdk1(4A)}$)

Y6035: *MAT* **a**, *pADH1-OsTIR1-myc*$_9$*::ADE2, SCC1-E2*$_6$*::K.l.TRP1, (HIS3MX6)pMET3-ECO1-IAA17::KAN*^{R}*MX6, pECO1-ECO1*$^{Q18A-L21A}$*-HA*$_3$*::URA3* (Eco1^{-pip})

Figure 1C

Y4396: *MAT* **a**, *pADH1-OsTIR1-myc*$_9$*::URA3 HOS1-IAA17::KAN*^{R}*MX6, SMC3-PK*$_3$*:HIS3MX6, pGAL1-ECO1-HA*$_3$*::LEU2*

Y4407: *MAT* **a**, *pADH1-OsTIR1-myc*$_9$*::URA3 HOS1-IAA17::KAN*^{R}*MX6, SMC3-PK*$_3$*:HIS3MX6, ECO1-HA*$_3$*::LEU2*

Figure S1A

DOM0077: *MAT* **a**, *bar1Δ::HISG, sic1Δ::LEU2, pRS426::pGAL1-CLB2-TAP::URA3* (2μ)

YSC3869-202333687: *MAT* **a**, *pep4-3, pGAL1-SWE1-HIS*$_6$*-HA-3C-ProtA* (2μ)

Figure S1B

Y6687: *MAT* **a**, *pep4Δ::HIS3MX6, wpl1Δ::LEU2, eco1Δ::KAN*^{R}*MX6, SCC1-E2*$_{6}$*::K.l.TRP1, pECO1-ECO1-HA*$_{3}$*::URA3*

Y6688: *MAT* **a**, *pep4Δ::HIS3MX6, wpl1Δ::LEU2, eco1Δ::KAN*^{R}*MX6, SCC1-E2*$_{6}$*::K.l.TRP1, pECO1-ECO1*^{S67A,T94A,S99AS,105A}*-HA*$_{3}$*::URA3*

Y6689: *MAT* **a**, *pep4Δ::HIS3MX6, wpl1Δ::LEU2, eco1Δ::KAN*^{R}*MX6, SCC1-E2*$_{6}$*::K.l.TRP1, pECO1-ECO1*^{Y164A}*-HA*$_{3}$*::URA3*

Y6690: *MAT* **a**, *pep4Δ::HIS3MX6, wpl1Δ::LEU2, eco1Δ::KAN*^{R}*MX6, SCC1-E2*$_{6}$*::K.l.TRP1, pECO1-ECO1*^{S98A,S104A}*-HA*$_{3}$*::URA3*

Figure S2

Y4798: *MAT* **a**, *pADH1-OsTIR1-myc*$_{9}$*::ADE2, SCC1-E2*$_{6}$*::K.l.TRP1, ECO1-HA*$_{3}$*::HIS3MX6, pGAL1-GAL4::URA3*

Y4788: *MAT* **a**, *pep4Δ::HIS3MX6, wpl1Δ::LEU2, eco1Δ::KAN*^{R}*MX6, SCC1-E2*$_{6}$*::K.l.TRP1, pGAL1-ECO1-HA*$_{3}$*::URA3, pGAL10-GAL4::ADE2*

Y4808: *MAT* **a**, *pep4Δ::HIS3MX6, wpl1Δ::LEU2, eco1Δ::KAN*^{R}*MX6, SCC1-E2*$_{6}$*::K.l.TRP1, pGAL1-ECO1-HA*$_{3}$*::URA3, pGAL10-GAL4::pGAL1-SCC1-HA*$_{3}$*::ADE2*

Strains for protein purification

Rfc1-RFC

Y6371: MAT **a**, *ade2-1, ura3-1, his3-11,15, trp1-1, leu2-3,112, can1-100, bar1::Hyg, pep4::KanMX, ura3::URA3-pRS306-RFC2+CBP-RFC3, trp1::TRP1-pRS304-RFC4 + RFC5, his3::HIS3-pRS303-RFC1+Gal4, ctf18Δ:: LEU2*

Ctf18-RFC

Y6079 MAT **a**, *ade2-1, ura3-1, his3-11,15, trp1-1, leu2-3,112, can1-100, bar1::Hyg, pep4::KanMX, ura3::URA3-pRS306-RFC2+CBP-RFC3, trp1::TRP1-pRS304-RFC4 + RFC5,*

his3::HIS3-pRS303-Ctf18-HA-PA+Gal4, leu2::LEU2-pRS305-CTF8+DCC1

Pds5

Y5629 MAT **a**, *ade2-1, ura3-52, his3-11,15, trp1-1, leu2-3,112, can1-100, pep4::HIS3, wpl1Δ::LEU2, eco1Δ::KanMX6, ade2::ADE2-pRSII402-Gal4-pGal1/10-Pds5-3E2a-PA*

Yeast strains for expression and purification of the other replication and cohesion factors were as previously described (Minamino et al., 2018; Posse et al., 2021).

Table S2. Oligonucleotide Sequences, Related to STAR Methods

Name	Sequence (5'-3')	Purpose
Y767	[bioTEG]GGTCTGACAGTTACCAATGCTTAATCA GTGAGGCACCTATCTCAGCGATCTGTCTATTTC GTTCATCCATAGTTGCCTGACTCCCCGTCGTGT AGATAAC	Primer extension/PCNA loading assay
MM256	CTAAATTGTAAGCGTTAATATTTTGTTAAAATTC GCGTTAAATTTTTGTTAAATCAGCTC	Primer extension/PCNA loading assay
MM211	TTGCGTATTGGGCGCTCTTCCGCTTCC	PCNA loading assay
CB408	GCAACGTTGTTGCCATTGCTACAG	Primer extension
long TH46	TCTTACTGTCATGCCATCCGTAAGATGCTTTTC TGTGACTGGTGAGTACTCAACCAAGTCA[BtndT] TCTGAGAATAGTGTATGCGGCGACCGAGTTGC TCTTGC	Primer extension/ internally biotinylated DNA
MM254A	TCGCCACTGGCAGCAGCCACTGGTAACAGGA TTA	Primer extension/ internally biotinylated DNA
CB296	AGTTGGGTAACGCCAGGGTTTTCCCAGTCAC GACG	Primer extension/ internally biotinylated DNA
MM314	GGTCGTGGCAAGCTGA	Introduction of a short and long gap
MM315	GGACAATTGACGCTGA	
MM316	GGTCATCTGAGCTGA	
MM317	GGCTACAATCTGCTGA	
MM318	GGCAGCTGCTGA	Introduction of a long gap
MM319	TCAGCTTGCCACGACCTCAGCGTCAATTGTCC TCAGCTCAGATGACCTCAGCAGATTGTAGCC (with or without 5' phosphate)	Filling in a small gap to obtain nicked or closed DNA
MM320	AGTTCGACGGTCAGCTTGCCACGACCTCAGCG TCAATTGTCCTCAGCTCAGATGACCTCAGCAGA TTGTAGCC	Filling in a small gap to obtain flapped DNA
CB630	Phospho-AGCTGCCTCAGCTTGCCACGACCTCA GCGTCAATTGTCCTCAGCTCAGATGACCTCAG CAGATTGTAGCCTCAGC	Nicking insert
CB631	Phospho-AGCTGCTGAGGCTACAATCTGCTGAG GTCATCTGAGCTGAGGACAATTGACGCTGAGG TCGTGGCAAGCTGAGGC	Nicking insert
